# Regulation of CHK1 inhibitor resistance by a c-Rel and USP1 dependent pathway

**DOI:** 10.1042/BCJ20220102

**Published:** 2022-10-14

**Authors:** Jill E. Hunter, Amy E. Campbell, Nicola L. Hannaway, Scott Kerridge, Saimir Luli, Jacqueline A. Butterworth, Helene Sellier, Reshmi Mukherjee, Nikita Dhillon, Praveen D. Sudhindar, Ruchi Shukla, Philip J. Brownridge, Hayden L. Bell, Jonathan Coxhead, Leigh Taylor, Peter Leary, Megan S.R. Hasoon, Ian Collins, Michelle D. Garrett, Claire E. Eyers, Neil D. Perkins

**Affiliations:** 1 Newcastle University Biosciences Institute, Faculty of Medical Sciences, Newcastle University, Newcastle Upon Tyne NE2 4HH, U.K.; 2 Centre for Proteome Research, Department of Biochemistry and Systems Biology, Institute of Systems, Molecular and Integrative Biology, University of Liverpool, Liverpool L69 7ZB, U.K.; 3 Newcastle University Clinical and Translational Research Institute, Preclinical In Vivo Imaging (PIVI), Faculty of Medical Sciences, Newcastle University, Newcastle Upon Tyne NE2 4HH, U.K.; 4 Bioinformatics Support Unit, Faculty of Medical Sciences, Newcastle University, Newcastle Upon Tyne NE2 4HH, U.K.; 5 Division of Cancer Therapeutics, The Institute of Cancer Research, Sutton SM2 5NG, U.K.; 6 School of Biosciences, Stacey Building, University of Kent, Canterbury, Kent CT2 7NJ, U.K.

**Keywords:** CHK1, CREl, deubiquitinase, DNA replication stress, inhibitor resistance, nuclear factor κB

## Abstract

Previously, we discovered that deletion of c-Rel in the Eµ-Myc mouse model of lymphoma results in earlier onset of disease, a finding that contrasted with the expected function of this NF-κB subunit in B-cell malignancies. Here we report that Eµ-Myc/*cRel^−/−^* cells have an unexpected and major defect in the CHK1 pathway. Total and phospho proteomic analysis revealed that Eµ-Myc/*cRel^−/−^* lymphomas highly resemble wild-type (WT) Eµ-Myc lymphomas treated with an acute dose of the CHK1 inhibitor (CHK1i) CCT244747. Further analysis demonstrated that this is a consequence of Eµ-Myc/*cRel^−/−^* lymphomas having lost expression of CHK1 protein itself, an effect that also results in resistance to CCT244747 treatment *in vivo*. Similar down-regulation of CHK1 protein levels was also seen in CHK1i resistant U2OS osteosarcoma and Huh7 hepatocellular carcinoma cells. Further investigation revealed that the deubiquitinase USP1 regulates CHK1 proteolytic degradation and that its down-regulation in our model systems is responsible, at least in part, for these effects. We demonstrate that treating WT Eµ-Myc lymphoma cells with the USP1 inhibitor ML323 was highly effective at reducing tumour burden *in vivo*. Targeting USP1 activity may thus be an alternative therapeutic strategy in MYC-driven tumours.

## Introduction

The Nuclear Factor κB (NF-κB) family of transcription factors, comprising RelA/p65, RelB, c-Rel, p50/p105 (NF-κB1) and p52/p100 (NF-κB2), are important regulators of cancer cell biology [1]. Through their ability to regulate a wide variety of genes associated with inflammation, proliferation, apoptosis and metastasis, aberrant NF-κB subunit activity can promote the growth, survival and spread of tumour cells [1]. In many haematological malignancies, mutations in the upstream regulators of the NF-κB pathway can lead to constitutive activation [2]. Consequently, NF-κB activity can promote the growth and survival of B-cell-like-diffuse large B-cell lymphomas (ABC-DLBCL), [3] primary mediastinal large B-cell lymphoma (PMBL) [4,5] and classical Hodgkin lymphoma (CHL) [6]. Recently, the RelB NF-κB subunit has been reported to confer resistance to DNA damage in DLBCL [7]. However, experimentally, the functions of specific NF-κB subunits have rarely been explored. Indeed, while there is often an assumption that NF-κB is an obligate tumour promoter, tumour suppressor-like characteristics have been identified *in vitro* that are rarely examined using *in vivo* models [1]. For example, in response to inducers of DNA replication stress, NF-κB can have a pro-apoptotic function [8–11].

Checkpoint kinase 1 (CHK1, CHEK1) plays a critical role in the response to DNA replication stress, which results from stalled DNA replication forks. In cancer cells, replication stress drives both genomic instability and clonal evolution [12–14]. It can be induced by a variety of mechanisms, including DNA damaging agents and by oncogenes such as MYC driving hyper-DNA replication [12–14]. Critical regulators of the cellular response to DNA replication stress not only include CHK1 but also the kinase Ataxia Telangiectasia and Rad3 Related (ATR), which protect against tumorigenesis through promoting DNA repair [14,15]. However, once established, tumour cells can also become addicted to this pathway since it enables them to survive on-going, potentially lethal, genomic instability. Therefore, inhibiting key protein kinases, such as CHK1, provides a potential therapeutic strategy that specifically targets tumours that have become reliant on their activity [16]. By inhibiting CHK1, or potentially other components of this pathway, tumour cells will accumulate non-survivable levels of DNA damage and ultimately die.

We and others have shown that there is significant cross-talk, between the ATR–CHK1 and NF-κB pathways. For example, phosphorylation of the putative CHK1 Thr 505 (T505) phosphosite in the RelA transactivation domain *in vitro* results in inhibition of tumour promoting activities of NF-κB, including resistance to apoptosis, autophagy, cell proliferation and cell migration [9–11,17–19]. Direct phosphorylation of the p50 NF-κB subunit on Ser 329 by CHK1 was demonstrated following DNA damage [20,21]. *In vitro* phosphorylation of p50 by CHK1 has been shown to regulate DNA binding of the p50 homodimer through phosphorylation on Ser 242, and homodimerisation through a phosphorylation event on Ser 337 [22]. Moreover, Kenneth et al. [23] found that the c-Rel NF-κB subunit controls the expression of Claspin in cancer cell lines. This is of particular relevance to investigating NF-κB's role in these pathways as Claspin is an adaptor protein associated with DNA replication forks that is required for ATR-dependent phosphorylation of CHK1 following DNA replication stress [24,25].

Deubiquitinases (DUBs) are a family of enzymes that act on ubiquitinated substrates to catalyse the removal of ubiquitin moieties [26]. One of the most well characterised DUBs is Ubiquitin-specific protease 1 (USP1). It is a key regulator of DNA repair, through for example, stabilising members of the DNA damage response, such as FANCD2 and PCNA by removing the Lys48-ubiquitin degradation signal [27,28]. There is an increasing body of evidence that the USP family of DUBs play important roles in tumourigenesis; some are reported to stabilise and regulate tumour suppressors, whilst others stabilise known oncogenes (reviewed in [29]). In much the same way that NF-κB can have both tumour promoting or suppressing roles depending on the cellular context [1,30–33], DUBs such as USP7 can elicit its effects by removing ubiquitin moieties from both tumour suppressor proteins such p53 [34] and oncogenes such as c-Myc [35]. Interestingly, USP1 has been described as an oncogene in Acute Myeloid leukaemia (AML) [30], and USP1 inhibition has been shown to reduce primary AML cell growth by promoting degradation of the ID1 protein and disrupting homologous recombination [36]. Other USP family members including USP7, USP9X and USP10, have been identified as potential therapeutic targets in various hematological malignancies (reviewed in [37]).

In three parallel manuscripts, including this one, we have used an integrated ‘omics-based approach to investigate how both *de novo* and acquired resistance to CHK1 inhibition develops, using cell line and mouse models [19,38]. Here we report that Eµ-Myc/*cRel^−/−^* cells have a major defect in the CHK1 pathway, which leads to therapeutic resistance to a highly specific CHK1i, CCT244747. This loss, or inactivation of the CHK1 pathway is mediated at least in part by down-regulation of the CHK1 DUB, USP1 and we propose that this represents a potential first step in the development of cellular resistance to such inhibitors. In a parallel report we use a mouse model where the RelA(p65) NF-κB subunit has been engineered to mutate the putative Thr505 CHK1 phosphosite to alanine [19]. In contrast with the results shown here, we find that although Eµ-Myc *RelA*^T505A^ mice also display resistance to CHK1 inhibition, they retain CHK1 protein. However, we find that Eµ-Myc *RelA*^T505A^ lymphomas also possess altered CHK1 activity. We show that the consequences of CHK1 inhibition *in vivo* are different from those seen in wild-type counterparts, with fewer and different phosphorylated proteins being affected. There we propose that reduced levels of *CLSPN* (Claspin), a regulator of CHK1 activity, is an important component of this effect. The focus of this manuscript and our report investigating resistance to CHK1 inhibition in the *RelA*^T505A^ mouse model [19], are the mechanisms that lead to defects in CHK1 activity. This removal or alteration of the target of the CHK1i is an important component in the development of resistance but is not the only change these cells need to undergo. In the final paper in this series, we bring the Eµ-Myc *RelA*^T505A^ and *c-Rel^−/−^* models together to consider how these lymphomas cope with these defects in CHK1 signalling [38]. We demonstrate that both models have up-regulated compensatory signalling pathways. Moreover, we show that Eµ-Myc *RelA*^T505A^ and *c-Rel^−/−^* lymphomas, while resistant to CHK1 inhibition are now sensitive to targeting these bypass pathways [38]. These results have implications for how CHK1i resistance might arise in human patients and, importantly, suggest potential combination or second line therapies to overcome this.

## Results

### Eµ-Myc*/c-Rel^−/−^* lymphoma cells are resistant to CHK1 inhibition

Over-expression of c-Myc is a feature of many types of cancer and results in DNA replication stress leading to genomic instability and tumorigenesis [14,15]. Therefore, the well-established Eµ-Myc mouse model of B-cell lymphoma [39] is an ideal system to explore how different NF-κB subunits might regulate these processes and potentially affect treatment with CHK1 inhibitors. We have previously found that knockout of the c-Rel NF-κB subunit in the Eµ-Myc model results in reduced survival [40]. We have also previously shown that the CHK1 inhibitor (CHK1i) SRA737, having just completed Phase I clinical trials (https://clinicaltrials.gov/ct2/show/NCT02797964), inhibits the growth of re-implanted wild-type (WT) Eµ-Myc cells [41]. Since c-Rel has been described as an indirect regulator of CHK1 activity by inducing CLSPN gene expression [23], we were curious as to whether deleting c-Rel would affect ATR/CHK1 signalling in Eµ-Myc lymphomas and consequently the response to CHK1 inhibition. We hypothesised that altered activation of CHK1 by ATR in response to MYC-induced DNA replication stress in Eµ-Myc/*cRel^−/−^* lymphoma cells could affect CHK1 inhibitor sensitivity.

**Figure 1.**
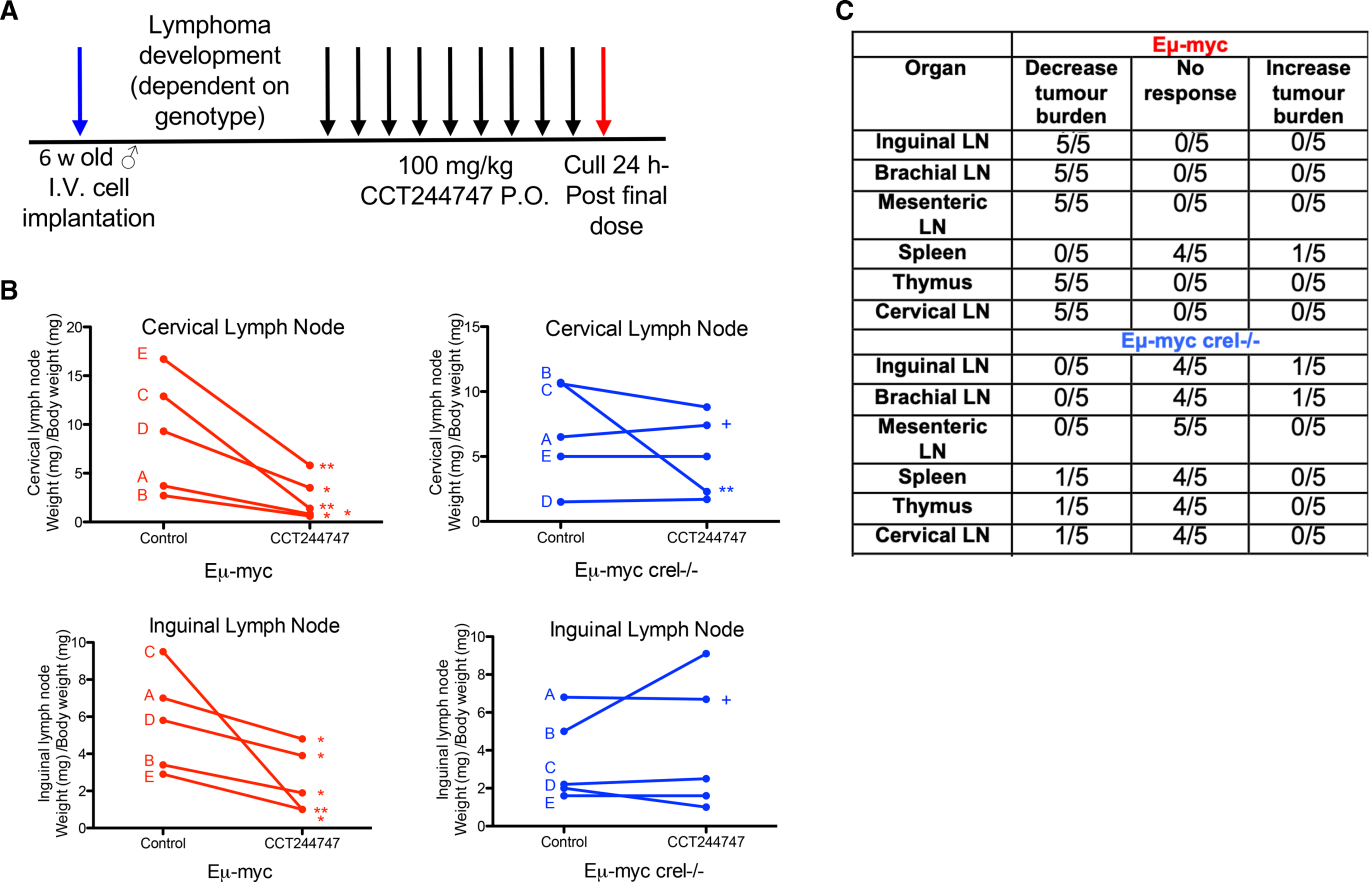
Eµ-Myc/*cRel^−/−^* lymphomas are resistant to Chk1 inhibition. (**A**) Schematic diagram illustrating the CHK1i *in vivo* study in Eµ-Myc and Eµ-Myc/*cRel^−/−^* mice. Six weeks old C57Bl/6 WT mice were implanted with either Eµ-Myc or Eµ-Myc/*cRel^−/−^* (blue arrow) and once tumours became palpable were treated with either 100 mg/kg CCT244747 p.o or vehicle control once daily for 9 days (black arrows). Mice were euthanised 24 h after the final dose (red arrow) and tumour burden assessed. (**B**) Line graphs showing the mean response of the five re-implanted Eµ-Myc and Eµ-Myc/*cRel^−/−^* (blue) tumours and their response to CCT244747. Each of the five spontaneously derived tumours was implanted into six syngeneic recipient C57Bl/6 mice, three were treated with CCT244747 (100 mg/kg p.o), and three with vehicle control, for 9 days once lymphoid tumours became palpable. A response was defined as a significant change in tumour burden (*P* < 0.05) using unpaired Student's *t*-tests. The complete data set is summarised in (**D**). ‘+’ indicates one experiment where treatment was stopped after 7 days and the mice were killed early due to the mice becoming too ill. (**C**) Table showing the response of five re-implanted Eµ-Myc and Eµ-Myc/*cRel^−/−^* tumours to CCT244747, in all sites where lymphoid tumour burden is anticipated in this model. Please note that the data from WT Eµ-Myc mice shown here is also used in our study on RelA T505A Eµ-Myc lymphomas [19]. These experiments were performed in parallel as part of the same larger study.

We therefore evaluated the effectiveness of the CHK1i CCT244747 (a selective CHK1 inhibitor with a similar reported *in vitro* profile to SRA-737 [41]) *in vivo* by analysing its effect on the growth of five transplanted WT Eµ-Myc and Eµ-Myc/*cRel^−/−^* tumours. Each tumour, which originated from an independently derived spontaneous tumour bearing mouse, was implanted via the lateral tail vein into six syngeneic C57Bl/6 recipient mice and three were treated orally with CCT244747 once a day for nine days, while three received a vehicle control (Figure 1A). Treatment commenced at the point at which tumours in the lymphoid organs became palpable. After treatment, we observed a striking reduction in lymphoid tumour burden in all mice re-implanted with WT Eµ-Myc lymphomas (Figure 1B,C, Supplementary Figure S1A). In contrast, four of the five Eµ-Myc/*cRel^−/−^* lymphomas showed no significant reduction in lymphoid tumour burden after CCT244747 treatment, with one lymphoma only exhibiting a partial response in the thymus and cervical lymph nodes. The resistance of Eµ-Myc/*cRel^−/−^* lymphomas was confirmed *ex vivo*. Treatment of Eµ-Myc lymphoma cells with CCT244747 for 96 h resulted in small but significant differences, with WT cells having reduced viability relative to Eµ-Myc/*cRel^−/−^* cells (Supplementary Figure S1B). Also included in this analysis were Eµ-Myc/*Rela*^T505A^ lymphoma cells, which we have shown elsewhere are also CCT244747 resistant [19]. The reduced magnitude of the effects of CCT244747 seen here likely reflects the low level of proliferation seen with Eµ-Myc cells when cultured *ex vivo*. These data confirmed that regulation of CHK1/DNA replication stress by the c-Rel NF-κB subunit *in vivo* significantly affects the sensitivity of Eµ-Myc lymphoma cells to CHK1 inhibition but the mechanism involved was not known.

### Eµ-Myc lymphomas lacking c-Rel exhibit altered cell signalling and response to CHK1 inhibition

As reported in Hunter et al. [19], CLPSN mRNA expression is significantly down-regulated in Eµ-Myc lymphoma cells either lacking c-Rel or that contain a phosphonull version of Thr 505 (T505A) on RelA [19]. We hypothesised therefore, that ATR/CHK1 signalling might be compromised in Eµ-Myc/*cRel^−/−^* lymphoma cells. Consequently, we decided to explore how these cells respond at an early time point to a single dose of CCT244747 *in vivo*. By examining this acute response, we reasoned that we could gain insights into how signalling in these cells had been rewired, something not possible with longer CCT244747 treatment where the mixture of dead, dying and surviving lymphoma cells was likely to confound analysis. We therefore investigated the nature of the response of re-implanted WT and Eµ-Myc/*cRel^−/−^* lymphomas following acute treatment with the CHK1i, CCT244747, using a combination of (phospho)proteomic and RNA Seq analysis, as described (Supplementary Figure S2A) [19].

To explore regulation of phosphorylation-mediated signalling pathways in these re-implanted lymphomas, we used tandem mass tag (TMT)-based isobaric labelling to quantify relative changes in both total protein levels and phosphopeptide abundance (Supplementary Figure S2). As reported in [19], of the ∼4000 proteins identified at a 1% false discovery rate (FDR), ∼2500 were quantified in at least three biological replicates (Supplementary Data File S1). At the phosphopeptide level, we identified over 6500 phosphopeptides, quantifying ∼3350 in at least three replicates (>4500 in at least two bioreps; Supplementary Data File S1). STRING analysis (https://string-db.org/) of the phosphoproteomic data from WT Eµ-Myc lymphomas confirmed effective targeting of CHK1 by CCT244747 *in vivo* [19].

**Figure 2.**
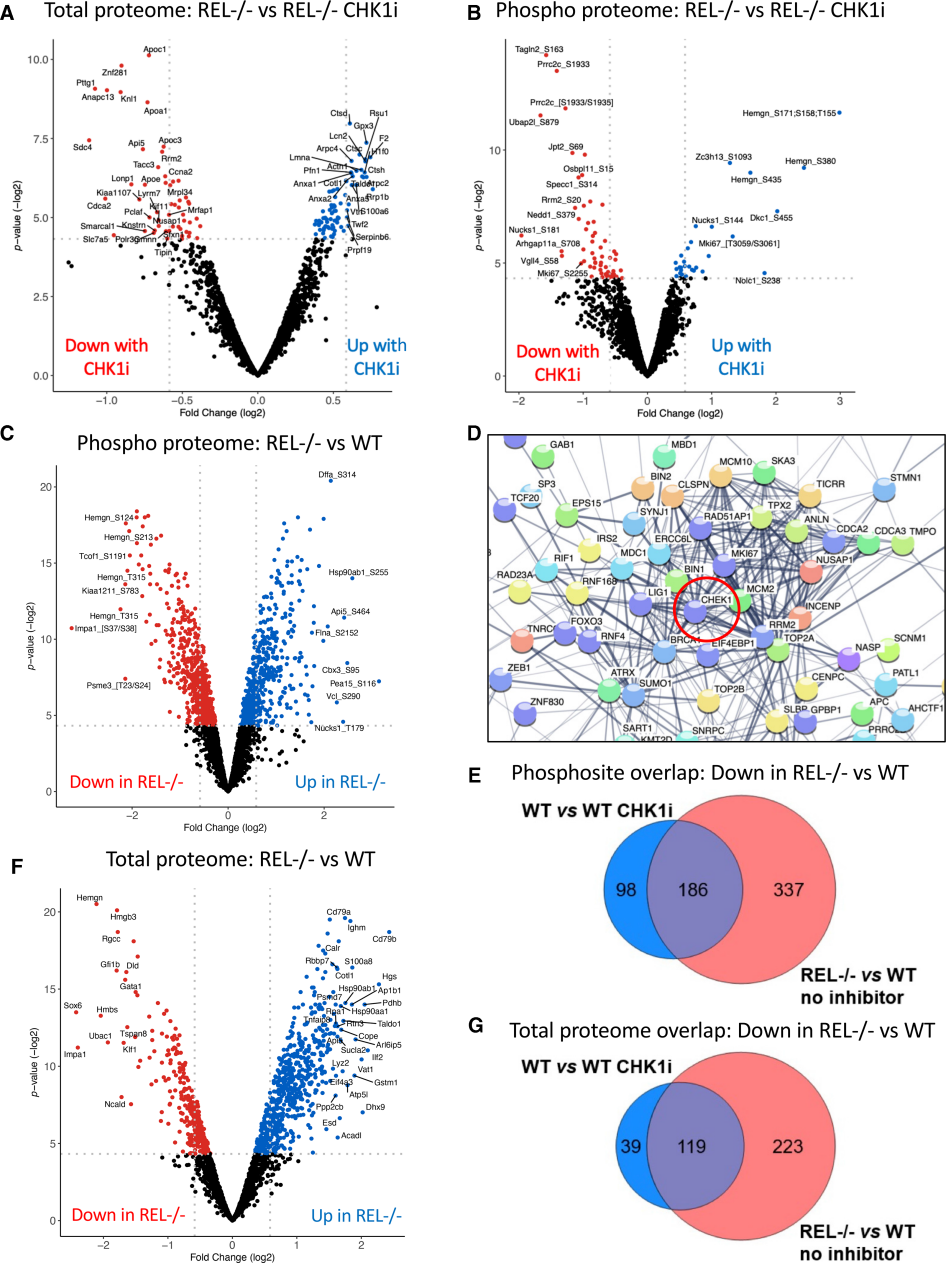
Proteomic analysis of WT Eµ-Myc and Eµ-Myc/*cRel^−/−^* lymphomas. (**A** and **B**) Volcano plots illustrating the significant number of CCT244747 effects in Eµ-Myc/*cRel^−/−^* lymphomas on both the total (**A**) and phospho (**B**) proteome. Down-regulation is shown with the red dots and up-regulation is shown with the blue dots. (**C**) Volcano plot demonstrating the significant number of phospho-proteomic differences between the Eµ-Myc WT and Eµ-Myc/*cRel^−/−^* lymphomas. Five hundred and eighty-nine down-regulated phospho-peptides (shown by the red dots were observed) and 517 up-regulated phospho-peptides (blue dots) in Eµ-Myc/*cRel^−/−^* tumours when compared with Eµ-Myc WTs. (**D**) STRING analysis of the proteins associated with the 589 down-regulated phospho-peptides in the Eµ-Myc/*cRel^−/−^* lymphomas revealed that many of these had known linkages with CHK1 or CHK1 signalling. Analysis performed under medium confidence setting. Please note that to illustrate the links to CHK1, this was added manually into the analysis (circled in red). However, since the string analysis was limited to only the query proteins, this does not increase the number of connections apart from those to CHK1 itself (see also Supplementary Figure S3D–F, Supplementary Data File S2). (**E**) Venn diagram illustrating that of the 284 unique down-regulated phosphosites seen in Eµ-Myc WT tumours following acute CCT244747 treatment, 186 were also down-regulated in Eµ-Myc/*cRel^−/−^* lymphoma cells without inhibitor treatment. Further analysis of the Eµ-Myc WT tumours following acute CCT244747 treatment can be found in Hunter, Campbell et al. [19]. (**F**) Volcano plot demonstrating the significant number of total protein differences between the Eµ-Myc WT and Eµ-Myc/*cRel^−/−^* lymphomas. Down-regulated proteins are shown with red dots and up-regulated proteins are shown with blue dots. (**G**) Venn diagram illustrating that of the 158 down-regulated proteins seen in Eµ-Myc WT tumours following acute CCT244747 treatment, 119 were also down-regulated in Eµ-Myc/*cRel^−/−^* lymphoma cells without inhibitor treatment.

Our analysis of this data demonstrated a significant number of CCT244747 effects in WT Eµ-Myc lymphomas, with 622 proteins and 625 phosphopeptides exhibiting a significant up- or down-regulation (*P*-value ≤0.05) (Supplementary Figure S2B,C, also shown in [19]). Strikingly, in comparison, relatively few significant changes were seen on the total and phospho proteomes following acute CCT244747 treatment of Eµ-Myc/*cRel^−/−^* lymphomas, with only 162 proteins and 89 phosphopeptides being significantly differentially regulated (*P*-value ≤0.05) (Figure 2A,B). This was consistent with the lack of effectiveness on lymphoma growth seen with long term CCT244747 dosing (Figure 1B,C).

### Eµ-Myc/*cRel^−/−^* lymphomas have intrinsically down-regulated the CHK1 pathway prior to inhibitor treatment

To better understand the underlying mechanistic basis that explains the relatively few significant (phospho)protein changes observed in Eµ-Myc/*cRel^−/−^* lymphomas in response to treatment with CCT244747, we compared the protein and gene expression profiles of re-implanted WT and Eµ-Myc/*cRel^−/−^* lymphomas in the absence of CCT244747 treatment. The proteomic data revealed that Eµ-Myc/*cRel^−/−^* lymphoma cells had substantially rewired their cell signalling pathways, with a high level of both down (589) and up-regulated (517) phosphopeptides compared with wild-type (Figure 2C, Supplementary Figure S3A). Furthermore, ∼75% of the protein level changes (and over 62% of the phosphorylation changes) that were induced in response to Chk1i in the WT Eµ-Myc lymphomas were also observed in the Eµ-Myc/*cRel^−/−^* samples with no treatment, suggesting that inhibition of Chk1 with CCT244747 may be working in part by modulating c-Rel-dependent processes (Supplementary Figure S3B,C, Supplementary Data File S1).

STRING analysis of proteins with down-regulated phosphopeptides in c-Rel lymphoma cells versus WT cells revealed that many have known connections to CHK1 (Figure 2D, Supplementary Figure S3D–F, Supplementary Data File S2). Moreover, of the 284 unique down-regulated phosphosites seen in wild-type cells upon CCT244747 treatment, 186 (65%) were also down-regulated in Eµ-Myc/*cRel^−/−^* lymphoma cells (Figure 2E, Supplementary Data File S3). Analysis of the total protein differences between re-implanted c-Rel*^−/−^* Eµ-Myc lymphomas and their wild-type counterparts, either with or without CCT244747 treatment revealed a similar trend. There were substantial total protein differences between WT and Eµ-Myc/*cRel^−/−^* lymphomas in the absence of CHK1 inhibition (Figure 2F). Notably, of the 966 proteins whose levels were statistically significantly different between WT and Eµ-Myc/*cRel^−/−^* lymphoma cells (*P*-value >0.05), the majority (65%, 624 proteins) were elevated (Supplementary Data File S1). Moreover, there was considerable overlap between those proteins observed to be down-regulated in WT cells upon treatment with CCT244747 and the cohort of proteins at comparatively lower levels in Eµ-Myc/*cRel^−/−^* lymphomas without CHK1i treatment (Figure 2G, Supplementary Data File S3). Interestingly, the magnitude of these changes seen in the CCT244747 treated wild-type cells was generally lower than that seen constitutively in Eµ-Myc/*cRel^−/−^* lymphomas (Supplementary Figure S4). These results demonstrated that Eµ-Myc/*cRel^−/−^* lymphomas have an intrinsic defect in CHK1 kinase signalling, comparable to the effect of inhibiting CHK1 in WT Eµ-Myc cells.

### Analysis of down-regulated phosphosites in Eµ-Myc *Rel^−/−^* lymphomas

Using this proteomic dataset, we analysed in more detail the nature of the down-regulated phosphosites in Eµ-Myc *Rel^−/−^* lymphomas and in WT Eµ-Myc lymphomas treated with CCT244747, identifying many proteins associated with the Cell Cycle and DNA damage responses (Supplementary Data File S4). Previously, Blasius et al. published a list of proteins phosphorylated by recombinant CHK1, engineered to use the ATP analogue N6-benzyl (N6B)-ATP, when added to human HeLa cell nuclear extract [42]. Cross referencing our dataset with this revealed remarkably little overlap. Only 16/156 proteins with down-regulated phosphorylation in WT Eµ-Myc lymphomas upon CCT244747 treatment were also seen in the dataset from Blasius et al. (Supplementary Data File S4). Of these, we could only find 1 identical phosphosite between these datasets, Clip1_S194 (S195 in human). When we looked at the proteins in common between CCT244747 treated WT Eµ-Myc lymphomas and *Rel^−/−^* Eµ-Myc, there were only 6/98 proteins also found in the Blasius et al. study, with no identical phosphosites (Supplementary Data File S4). From their data, Blasius et al. [42] also derived a consensus motif for the CHK1 phosphosites they identified of R/K_x_x_S/T_F/Q. Of the putative common phosphopeptides between CCT244747 treated WT Eµ-Myc lymphomas and *Rel^−/−^* Eµ-Myc, where we could confidently predict the site of phosphorylation, there were only three that contained an SF, SQ or TQ motif (one of each).

These differences could arise from the very different approaches taken. It might be expected that phosphosites identified from the addition of recombinant CHK1 to a HeLa cell nuclear extract would be different from analysis of whole cell lysates extracted from a mouse B-cell lymphoma. In addition to the altered range of proteins expressed, other factors such as targeting of endogenous CHK1 to substrates *in vivo* via scaffold or accessory proteins may also be a factor. Indeed, in our parallel study examining Eµ-Myc *RelA^T505A^* lymphomas we find that the phosphopeptides altered upon CCT244747 treatment show significant differences to those observed in WT Eµ-Myc lymphomas [19]. However, we cannot rule out that the phosphosites we have identified are not direct CHK1 targets but rather the downstream consequences of CHK1 inhibition on other kinases. Nonetheless, these still provide a phospho-signature of the consequences of loss of CHK1 activity. Moreover, many of these are in proteins known to be associated with CHK1 activity, such as Claspin and BRCA1 (Supplementary Data File S4).

We also analysed the phosphoproteomic dataset from Eµ-Myc *Rel^−/−^* lymphomas for evidence of any general changes in ATR, Ataxia Telangiectasia Mutated (ATM) or DNA-Dependent Protein Kinase (DNA-PK, PRKDC) dependent phosphorylation, whose target phosphosites generally contain an SQ or TQ motif [43]. (Supplementary Data File S4). In total, we detected 144 phosphopeptides where we could confidently assign a phosphosite to an SQ or TQ motif. Functional annotation clustering of these using David (https://david.ncifcrf.gov/) revealed enrichment for GOTERMS including ‘Chromosome’, ‘DNA repair’, ‘DNA Damage’ and ‘Cell Cycle’, suggesting they represented likely targets for ATR or ATM signalling in Eµ-Myc lymphomas (Supplementary Data File S4). However, of these 144 phosphopeptides only 27 showed a significant difference (*P* < 0.05) between *Rel^−/−^* and WT Eµ-Myc lymphomas (no CCT244747 treatment). Of these 27, 11 were down-regulated in Eµ-Myc *Rel^−/−^* lymphomas and 16, similar to RPA2 Ser 33 (see below), were up-regulated (Supplementary Data File S4). Further functional annotation analysis of this set of proteins similarly revealed enrichment for the terms ‘Chromosome’, ‘DNA Repair’ and ‘Cell Cycle’ but phosphopeptides containing the associated SQ and TQ motifs were again observed as a mixture of both up and down-regulated responses (Supplementary Data File S4). Overall, this suggests no major disruption of signalling by ATR and ATM in Eµ-Myc *Rel^−/−^* lymphomas but that specific targets exhibit changes in phosphorylation that could impact on the phenotype of these cells.

### Analysis of RNA Seq data from Eµ-Myc lymphoma cells

**Figure 3.**
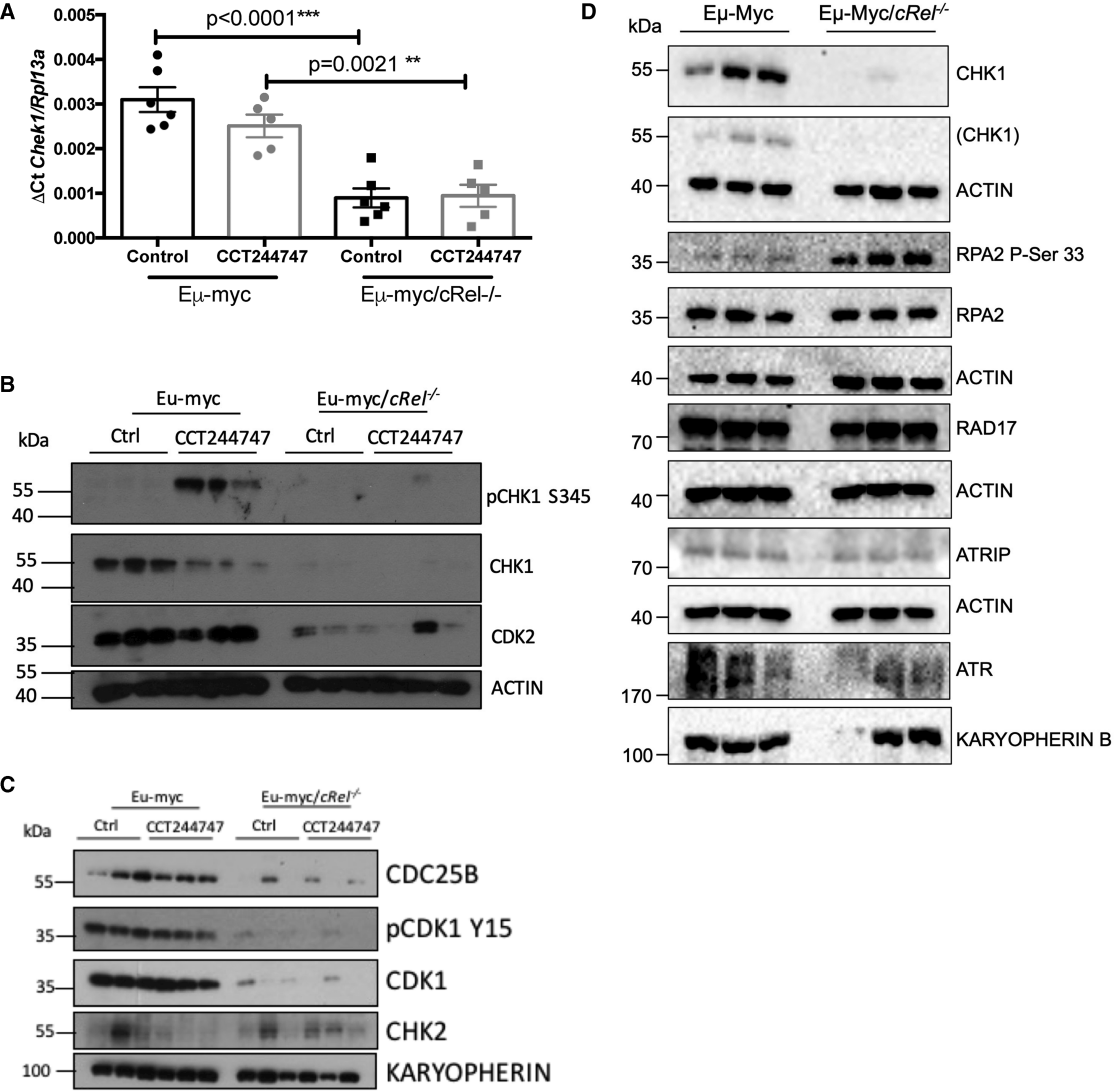
Loss of CHK1 expression in Eµ-Myc/*cRel^−/−^* lymphomas. (**A**) Q-PCR validation of RNA-Seq analysis. Relative CHEK1 transcript levels are significantly reduced in tumours from Eµ-Myc/*cRel^−/−^* (*n* = 6) when compared with Eµ-Myc WTs (*n* = 6). Data represents mean ± SEM. ** *P* < 0.01, *** *P* < 0.001 (One-way ANOVA with Tukey's post-hoc test). CHEK1 expression is also partially reduced in WT tumours following CCT244747 treatment. Data represents mean ± SEM, each point is an individual mouse. (**B**) Western blot analysis of phospho-Ser345 CHK1, CHK1, CDK2 or ACTIN in snap frozen tumour extracts prepared from re-implanted Eµ-Myc and Eµ-Myc/*cRel^−/−^* tumours mouse inguinal lymph nodes 8 h following a single dose of CCT244747. The expression of CHK1 and related pathway components are lost in Eµ-Myc/*cRel^−/−^* tumours. Please note the actin blot used here is replicated in another paper [19], where it is used as the control for CLSPN expression also analysed using this membrane. (**C**) Western blot analysis of CDC25B, phospho-Tyr15 CDK1, CDK1, CHK2 or KARYOPHERIN in snap frozen tumour extracts prepared from re-implanted Eµ-Myc and Eµ-Myc/*c-rel^−/−^* tumours mouse inguinal lymph nodes 8 h following a single dose of CCT244747. CDC25B, phospho-Tyr15 CDK1, CDK1, expression is lost in Eµ-Myc/*cRel^−/−^* tumours. (**D**) Western blot analysis of CHK1, RPA2 phospho Ser 33, total RPA2, RAD17, ATRIP and ATR using snap frozen tumour extracts prepared from re-implanted Eµ-Myc and Eµ-Myc/*c-Rel^−/−^* tumours mouse inguinal lymph nodes. ACTIN and KARYOPHERIN B were used as loading controls as indicated. The ACTIN control where the original CHK1 blot was reprobed has been expanded to show the position of the residual CHK1 signal.

We next analysed RNA Seq data to obtain further insights into the intrinsic transcriptional reprogramming of Eµ-Myc/*cRel^−/−^* lymphoma cells that leads to these proteomic and cell signalling alterations (Supplementary Data Files S5, S6). Functional profiling of the genes whose mRNA expression varied between WT and *c-Rel^−/−^* Eµ-Myc cells, revealed that of the 36 genes associated with ‘Activation of ATR in response to replication stress’ (REAC:R-HSA-176187), 32 (89%) were down-regulated in Eµ-Myc/*cRel^−/−^* cells (Supplementary Figure S5A). This included transcript levels of CHEK1, which we subsequently validated using qPCR (Figure 3A). However, of the 342 proteins whose levels were decreased in Eµ-Myc/*cRel^−/−^* cells compared with their wild-type counterparts, 123 (36%) were not also down-regulated at the transcript level, suggesting that there are also significant post-transcriptional effects on protein expression (Supplementary Figure S5B, Supplementary Data File S3). Western blot analysis confirmed not only that signalling through CHK1 was impaired in *c-Rel^−/−^* Eµ-Myc cells, but that there was almost complete loss of CHK1, CDC25B, CDK1 and CDK2 protein. (Figure 3B,C, and Supplementary Figure S5C). However, despite these perturbations in the levels of cell cycle regulatory proteins, no differences in cell cycle phase distribution were observed between of Eµ-Myc WT and Eµ-Myc/cRel*^−/−^* lymphoma cells (Supplementary Figure S5D). This suggests that, either the remaining levels of these cell cycle regulatory proteins are sufficient, or that other compensatory mechanisms exist.

We also observed loss of CLSPN in these extracts [19], in agreement with our proteomics data which revealed ∼1.6-fold lower levels (*P*-value = 5.36 × 10^−4^). Levels of the checkpoint kinase CHK2, which functions downstream of ATM in response to double strand DNA breaks, appeared broadly comparable, albeit variable, in the untreated Eµ-Myc WT and Eµ-Myc/cRel*^−/−^* lymphoma cells. However, after CCT244747 treatment, there was an apparent loss of CHK2 protein in WT Eµ-Myc cells, not seen in the Eµ-Myc/cRel*^−/−^* lymphomas (Figure 3C).

We also investigated the levels of other components of the CHK1 pathway, ATR, ATRIP and RAD17. In contrast, the Eµ-Myc/*cRel^−/−^* cells retained expression of these proteins (Figure 3D). As part of this experiment, using the same protein extracts, we examined phosphorylation of Replication Protein A (RPA) 2 (also known as RPA32) at serine 33 a marker for ATR activation and DNA replication stress [44]. RPA is a eukaryotic ssDNA-binding protein that is essential for DNA replication and repair [45]. It is not only crucial for the recruitment and activation of ATR but is also an ATR target [44,46]. In response to genotoxic stress, RPA2 is phosphorylated on Ser 33 by ATR and this phosphorylation subsequently stimulates further phosphorylation by Cyclin-CDKs and DNA-PK to yield hyperphosphorylated RPA [47]. RPA2 is also a target for ATM [48]. Levels of phosphorylation at this site were significantly increased in the Eµ-Myc/*cRel^−/−^* cells lymphomas, consistent with these cells experiencing high levels of DNA replication stress associated with loss of CHK1 protein.

Taken together, these data suggest that the *de novo* resistance of the Eµ-Myc/*cRel^−/−^* lymphoma cells to CCT244747 arises from these cells already having down-regulated the CHK1 pathway. Consequently, further attempts to inhibit CHK1 have little effect.

### Acquired resistance to CHK1 inhibition in U2OS cells is also associated with down-regulation of CHK1 protein

**Figure 4.**
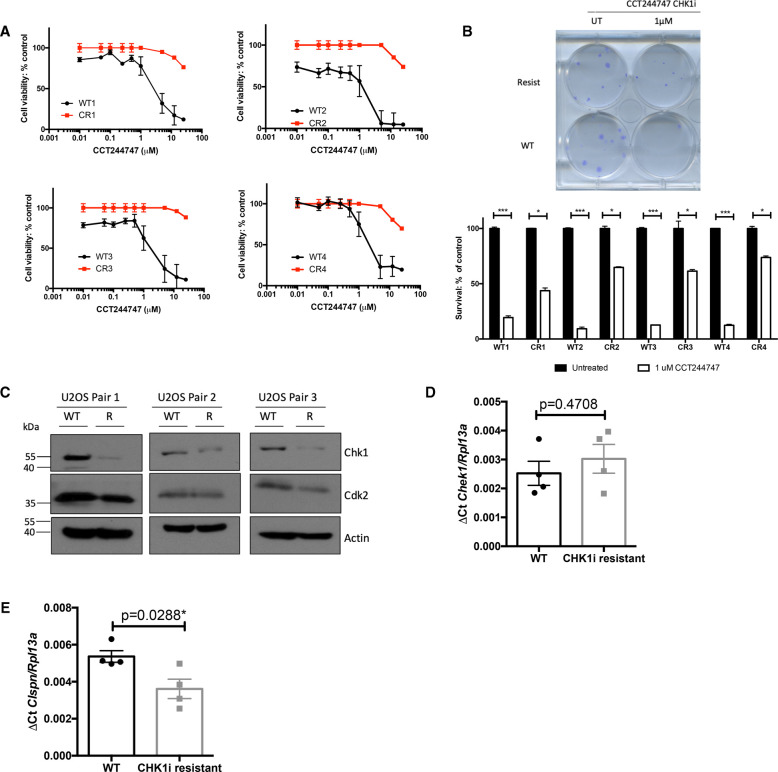
Down-regulation of CHK1 expression in CCT244747 resistant U2OS cells. (**A**) Four independently derived CCT244747 resistant (CR) U20S cell lines are resistant to CHK1 inhibitor treatment. Cell viability (Prestoblue assay) in WT and CR U20S following treatment with increasing concentrations of the CHK1 inhibitor, CCT244747 for 72 h. (**B**) Increased clonogenic survival in four independently derived CCT244747 resistant (CR) U20S cell lines following CHK1 inhibitor treatment. Representative image and bar graph data showing clonogenic survival in WT and CR U20S following either treatment with 1 µM CCT244747 or solvent controls for 24 h. Data was analysed using One-way ANOVA with multiple comparisons and Sidak's post-hoc test. *P*-values of *P* < 0.05 were considered signiﬁcant. (**C**) Western blot analysis of CHK1, CDK2, or ACTIN in extracts prepared from WT and CCT244747 resistant (CR) U20S. (**D**) Q-PCR data showing relative CHEK1 expression in four independently derived CCT244747 resistant (CR) U20S cell lines, or their WT counterparts. CHEK1 expression is unaffected in CR U20S. Data represents mean ± SEM, each point is the mean of three independent experiments in each of the four cell lines. Data was analysed using an Unpaired Student's *t*-test. *P*-values of *P* < 0.05 were considered signiﬁcant hence these data suggest no difference in *CHEK1* transcript levels. (**E**) Q-PCR data showing relative Claspin expression in four independently derived CCT244747 resistant (CR) U20S cell lines, or their WT counterparts. Claspin expression is reduced in CR U20S. Data represents mean ± SEM, each point is the mean of three independent experiments in each of the four cell lines * *P* < 0.05 (Unpaired Student's *t*-test).

We next wished to determine if similar effects on CHK1 levels and activity were seen as a consequence of acquired CHK1 inhibitor resistance. To this end, we generated four independent isolates of the osteosarcoma cell line, U2OS, with resistance to the CHK1i, CCT244747. This was achieved through long term culture in increasing concentrations of CCT244747. Eventually, the resistant U2OS cells were able to proliferate in high CCT244747 concentrations (Figure 4A) and retain clonogenic potential (Figure 4B). As controls we also passaged U2OS cells in the absence of CCT244747 to mimic effects of long-term culture.

**Figure 5.**
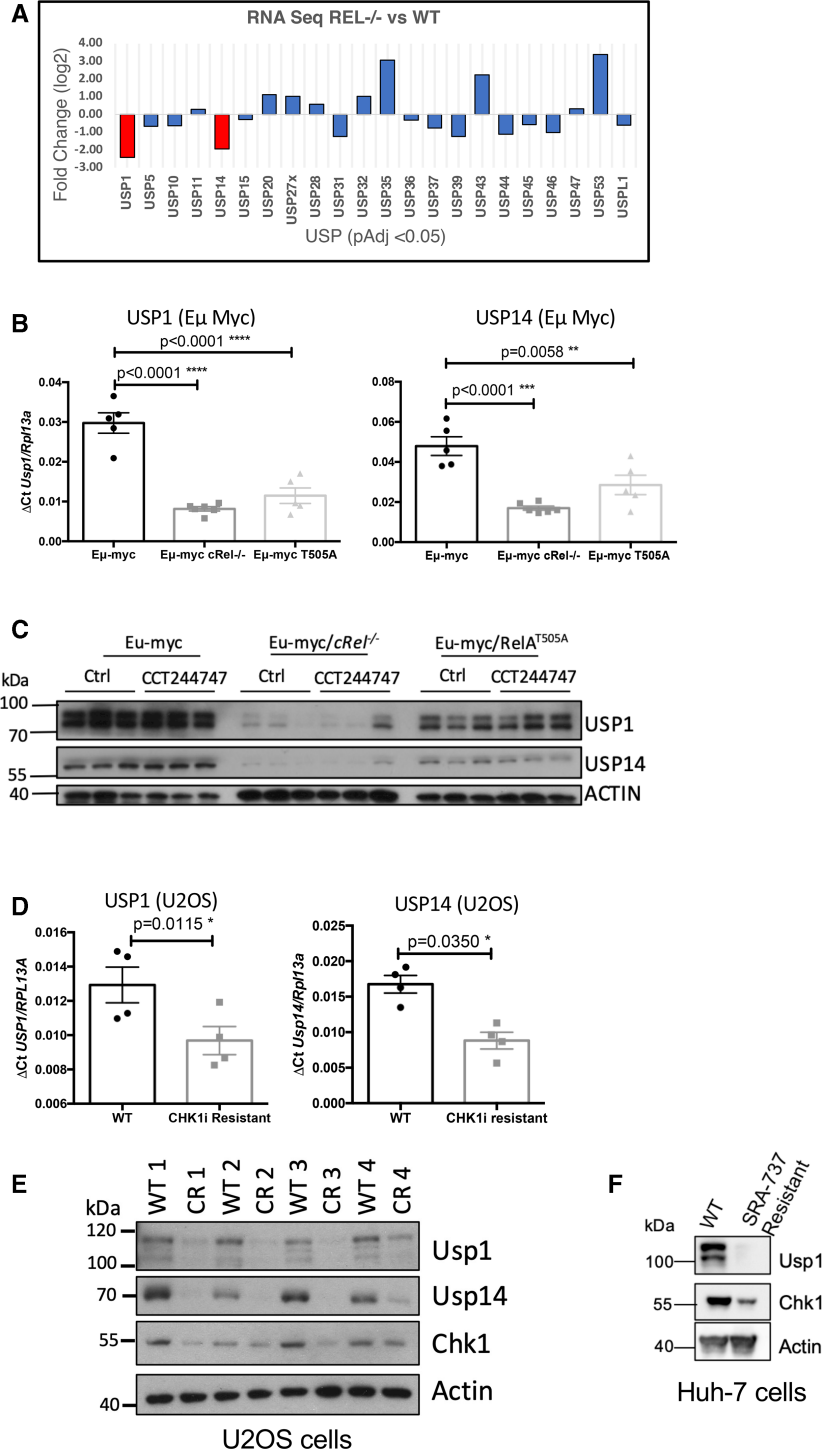
Down-regulation of CHK1 expression in CCT244747 resistant U2OS and Eµ-Myc cells. (**A**) Bar graph showing the relative expression of 24 DUBs that were significantly up- or down-regulated in the Eµ-Myc/*cRel^−/−^* tumours by RNA-Seq analysis. The red bars show that both USP1 and USP14 were down-regulated by ∼2-fold compared with Eµ-Myc WTs. (**B**) Q-PCR validation of RNA-Seq analysis. Relative USP1 and USP14 transcript levels are significantly reduced in tumours from Eµ-Myc/*cRel^−/−^* (*n* = 6) and Eµ-Myc/*RelA^T505A^* (*n* = 5) when compared with Eµ-Myc WTs (*n* = 5). Data represents mean ± SEM. ** *P* < 0.01, *** *P* < 0.001 (Unpaired student's *t*-test). Data represents mean ± SEM, each point is an individual mouse. (**C**) Western blot analysis of USP1, USP14 or ACTIN in snap frozen tumour extracts prepared from re-implanted Eµ-Myc, Eµ-Myc/*cRel^−/−^* and and Eµ-Myc/*RelA^T505A^* tumours mouse inguinal lymph nodes 8 h following a single dose of CCT244747. USP1 and USP14 expression is lost in Eµ-Myc/*cRel^−/−^* tumours and reduced in and Eµ-Myc/*RelA^T505A^* tumours. Please note that the Actin blot from this figure is also used in another study (Supplementary Figure S2C middle panel, [38]), where the same membrane was probed with antibodies to other proteins. (**D**) Q-PCR data showing relative USP1 and USP14 transcript levels are significantly reduced in in four independently derived CCT244747 resistant U20S cell lines, compared with WT U20S cells. Data represents mean ± SEM. * *P* < 0.05 (Unpaired student's *t*-test). Data represents mean ± SEM, each point is the mean of three independent experiments in each of the four cell lines. (**E**) Western blot analysis of USP1, USP14, CHK1, or ACTIN in extracts prepared from WT and CCT244747 resistant U20S. USP1 and USP14 expression is lost in CCT244747 resistant U20S. (**F**) Western blot analysis of USP1, CHK1, or ACTIN in extracts prepared from WT and SRA-737 resistant Huh-7 cells. USP1 expression is lost in SRA-737 resistant Huh-7 cells.

To determine whether CHK1 signalling was affected during the acquisition of resistance, we performed western blotting and qPCR analyses. Western blot analysis confirmed that CHK1 levels were reduced in three out of four CCT244747 resistant isolates (Figures 4C, 5E). However, by contrast with our data from Eµ-Myc/*cRel^−/−^* lymphoma cells, there was no reduction in CHK1 mRNA levels as determined by RNA Seq and qPCR analysis (Figure 4D, Supplementary Data Files S7, S8). We also observed a slight but significant reduction in Claspin transcript levels in CHK1i resistant U2OS cells (Figure 4E), mirroring the observations in our resistant mice [19]. We also failed to observe a reduction in expression of the 32 genes associated with ‘Activation of ATR in response to replication stress’ (>2 fold change, adj *P*-value <0.05; REAC:R-HSA-176187) that were down-regulated in the Eµ-Myc/*cRel^−/−^* cells. The exception to this was again CLSPN, where the RNA Seq data confirmed a 2.2-fold down-regulation (*P*_Adj_ value = 0.0037) in CCT244747 resistant U2OS cells (Supplementary Data Files S7, S8). Since we had observed potential differences in CHK2 levels in Eµ-Myc/*cRel^−/−^* lymphoma cells (Figure 3C), we investigated whether these CCT244747 resistant U2OS cells acquire sensitivity to CHK2 inhibition. As expected, WT cells showed a strong induction of γH2AX upon CC244747 treatment that was not seen in the CHK1i resistant cell lines. Moreover, we also observed a higher basal γH2AX signal in the CCT244747 resistant lines that would be consistent with a higher level of DNA replication stress concomitant with loss of CHK1. However, neither the wild-type nor the CCT244747 resistant lines showed an increase in γH2AX upon treatment with the CHK2i CCT241533 (Supplementary Figure S6A).

Taken together, the data suggest a consistent mechanism of both *de novo* and acquired resistance, namely down-regulation of CHK1 protein levels and thus activity, thereby rendering cells insensitive to a CHK1 inhibitor.

### Deregulation of ubiquitin mediated proteolysis in Eµ-Myc/*cRel^−/−^* lymphoma cells

Results from our Eµ-Myc lymphoma cell proteomic analysis above, together with the loss of CHK1 protein but not mRNA in the CCT244747 resistant U2OS cells, suggested that post-transcriptional regulation of protein levels was a key factor in acquisition of both *de novo* and acquired CHK1 inhibitor resistance. We therefore further analysed our RNA Seq data from wild-type and Eµ-Myc/*cRel^−/−^* lymphoma cells. Of the genes associated with ubiquitin-dependent proteolysis we observed a number of changes. Most strikingly, there was significant down-regulation of the deubiquitinases (DUBs) USP1 and USP14 (Figure 5A, Supplementary Data Files S5, S6). USP1 has been reported as a key regulator of DNA repair and is known to play a role in stabilising members of the DNA damage response, such as FANCD2 and PCNA [27,28] by removing the K48 ubiquitin degradation signal. Interestingly, one report suggested that USP1 can act as a DUB for CHK1, by protecting it from proteasomal degradation [49]. USP14 is often overexpressed in tumours and has been shown to deubiquitinate and stabilise the androgen receptor in models of breast and prostate cancer [50,51]. Down-regulation of these genes was validated by qPCR (Figure 5B), while western blot analysis revealed almost total loss of these proteins in extracts prepared from Eµ-Myc/*cRel^−/−^* lymphoma cells (Figure 5C, Supplementary Figure S6B). We also analysed samples from our Eµ-Myc/*RelA^T505A^*lymphoma cells that also display CCT244747 resistance [19] and found reduced levels of USP1 and USP14 mRNA and protein, albeit less dramatically than seen with loss of c-Rel (Figure 5C, Supplementary Figure S6B). In addition, both USP1 and USP14 mRNA and protein levels were lower in the U2OS CCT244747 resistant cells (Figure 5D,E). To further support these data, we analysed an additional cell line, Huh7 hepatocellular carcinoma cells, that had been generated to display resistance to the CHK1i, SRA-737. Consistent with our previous observations above, these cells also exhibited loss of both USP1 and CHK1 protein (Figure 5F). Consistent with the loss of USP1 protein in CCT244747 resistant U2OS cells, a clonogenic survival assay revealed that these cells had also acquired resistance to the USP1 inhibitor ML323 (Supplementary Figure S6C).

**Figure 6.**
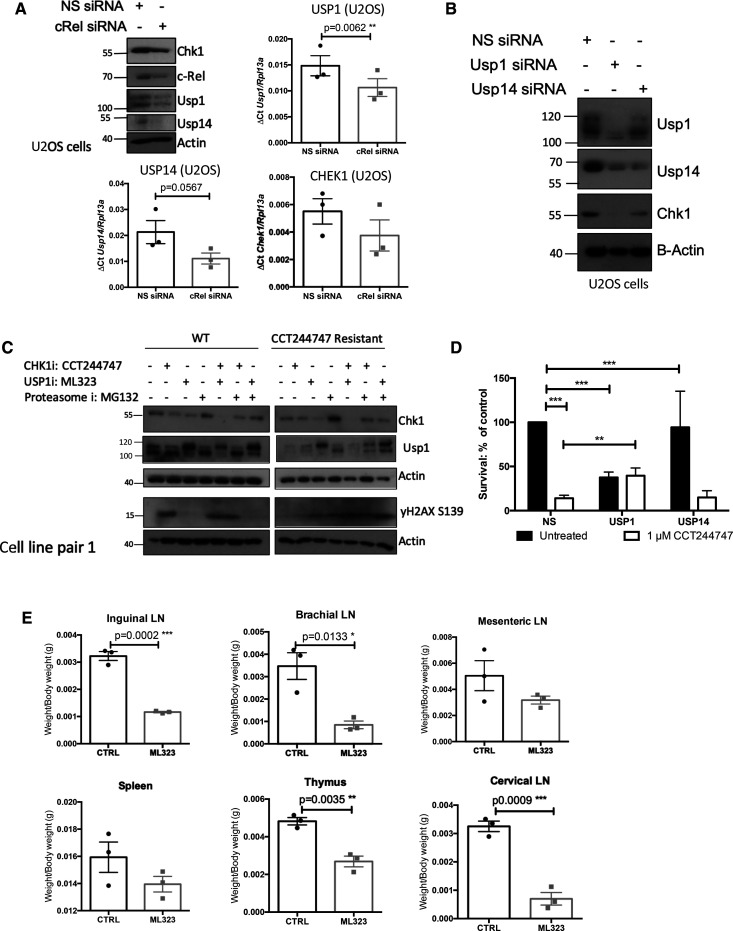
Loss of USP1 leads to down-regulation of CHK1 protein levels and acquisition of CCT244747 resistance. (**A**) Western blot and Q-PCR analysis from WT U2OS cells following siRNA targeting c-Rel or a Non-specific siRNA control. Western blot analysis shows that knockdown of c-Rel results in a reduction in USP1, USP14 and CHK1. Actin is used as a loading control. Q-PCR data shows that USP1 and USP14 transcript levels are reduced following c-Rel knockdown, but that CHEK1 transcript levels are unaffected. Data represents mean ± SEM, each point is the mean of three independent experiments. ** *P* < 0.01 (Unpaired student's *t*-test). (**B**) Western blot analysis from WT U2OS cells following siRNA targeting USP1, USP14 or a Non-specific siRNA control. Data shows that CHK1 is completely lost following USP1 knockdown and partially lost following USP14 knockdown. Actin is used as a loading control. (**C**) Western blot analysis of WT or CCT244747 resistant U2OS cells treated with CCT244747, the USP1 inhibitor ML323, or the Proteasome inhibitor MG-132, alone or in combination. Blots were probed for CHK1, USP1, yH2AX or Actin. Inhibition of USP1/14 in WT U2OS results in the loss of CHK1. Proteasomal inhibition in the CCT244747 resistant U2OS cells results in the stabilisation of CHK1 protein. (**D**) Clonogenic survival in WT U20S cell lines following siRNA targeting USP1, USP14 or a Non-specific siRNA control. U2OS cells are sensitive to CCT244747 in the presence of control or USP14 siRNA, however knockdown of USP1 renders them insensitive to CCT244747 treatment. Data represents mean ± SEM, each point is the mean of three independent experiments. *** *P* < 0.01 (One-way ANOVA with Tukey's post-hoc test). (**E**) Scatter showing the response of one re-implanted Eµ-Myc tumour to ML323 in the lymphoid tumour sites. One Eµ-Myc tumour was implanted into six syngeneic recipient C57Bl/6 mice, three were treated with ML323 (10 mg/kg i.p), and three with vehicle control, for 9 days once tumours became palpable. A response was defined as a significant reduction in tumour burden (*P* < 0.05) using unpaired Student's *t*-tests. WT Eµ-Myc tumour burden was reduced by ML323 treatment in all lymphoid tissues.

siRNA depletion of c-Rel in wild-type U2OS cells resulted in a reduction in both USP1 and USP14 mRNA and protein and this was associated with down-regulation of CHK1 protein but not CHK1 mRNA (Figure 6A). This suggests a conserved mechanism through which c-Rel can directly or indirectly control the transcription of USP1 and USP14, with the loss of one or both of these DUBs then resulting in CHK1 protein destabilisation.

### USP1 regulates CHK1 protein levels and mediates resistance to CHK1 inhibition

To determine whether USP1 or USP14 were responsible for CHK1 protein stability in our model, we used siRNAs to deplete levels of these proteins in WT U2OS cells. Loss of USP1 resulted in almost total loss of CHK1 at the protein level, suggesting that in the absence of this DUB, CHK1 is targeted by the proteasome for degradation (Figure 6B). Depletion of USP1 also reduced USP14 levels, while the USP14 siRNA resulted in partial loss of CHK1, suggesting that the activity of these DUBs may be linked. This was confirmed by treatment of WT U2OS cells with the USP1 inhibitor ML323 [52], which also resulted in a reduction in CHK1 protein levels (Figure 6C, Supplementary Figure S6D). Importantly, proteasome inhibition with MG132 restored CHK1 protein in the resistant U2OS cells (Figure 6C, Supplementary Figure S6D), and this in turn induced a DNA damage response as determined by elevated yH2AX phosphorylation, suggesting a potential restoration of CHK1i sensitivity. Interestingly, although there was some variability between the cell lines, while treatment of the resistant U2OS cells with ML323 and CCT244747 alone did not induce γH2AX S139 phosphorylation, using them in combination did (Figure 6C, Supplementary Figure S6D). The reason for this is unclear but suggests that the residual levels of these proteins in the CCT244747 resistant U2OS cells (Figure 5C, Supplementary Figure S6B) may functionally compensate for each other.

To determine whether loss of either USP1 or USP14, was responsible for the resistance to CHK1 inhibitors, we performed clonogenic assays following knockdown of either USP1 or USP14 in combination with CCT244747 treatment. Although loss of USP1 itself reduced the clonogenic potential of U2OS cells, the remaining cells now exhibited complete resistance to CHK1 inhibition (Figure 6D). In contrast, depletion of USP14 U2OS cells had little effect on either clonogenic potential or CCT244747 sensitivity (Figure 6D).

These data indicate that loss of USP1 can contribute to CHK1 inhibitor resistance.

### Inhibition of USP1 is a potential therapeutic strategy in cells with highly active CHK1

Given our finding that USP1 is highly abundant in the WT Eµ-Myc lymphoma cells (Figure 5C, Supplementary Figure S6B), together with our data suggesting that USP1 can control CHK1 proteasomal degradation, we hypothesised that targeting USP1 might represent a viable therapeutic strategy in tumours with high levels of genomic instability and replication stress. We therefore evaluated the effectiveness of the USP1/UAF inhibitor, ML323 *in vivo* [52–54]*,* and analysed its effect on the growth of transplanted WT Eµ-Myc tumours. As previously performed with CCT244747 (Figure 1), each tumour was implanted into six syngeneic C57Bl/6 recipient mice and three were treated intraperitoneally with ML323 once a day for nine days, while three received a vehicle control (Figure 6E). After treatment, we observed a striking reduction in lymphoid tumour burden in all mice treated with ML323. (Figure 6E). These data confirmed that highly active USP1 could be exploited therapeutically in tumours with on-going oncogene-induced replication stress.

## Discussion

**Figure 7.**
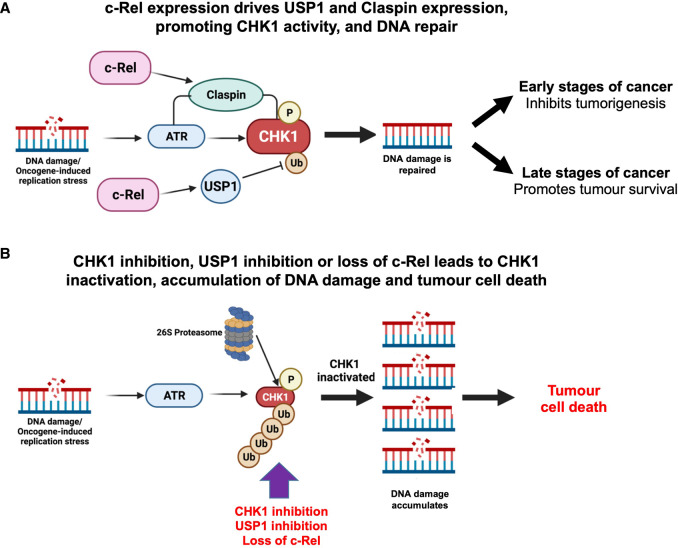
Model summarising the pathway linking c-Rel, USP1 and CHK1 in cancer cells. (**A**) DNA replication stress leads to activation of the ATR/CHK1 pathway that promotes DNA repair and genomic stability. c-Rel promotes the activity of this pathway by regulating the expression of the deubiquitinase USP1, which stabilises CHK1 protein, as well as the adaptor protein Claspin [19] which acts to promote CHK1 activation by ATR. At earlier stages of cancer or in normal cells, this pathway will help prevent the acquisition of further mutations leading to malignant tumour development. However, at later stages of cancer, tumours become addicted to this pathway as it helps them survive on-going high levels of DNA replication stress and genomic instability. (**B**) Since tumours become addicted to ATR/CHK1 signalling to help them survive high levels of genomic instability, inhibiting this pathway is an attractive anti-cancer strategy. This can be achieved through the use of CHK1 inhibitors such as SRA737 and CCT244747. We propose that USP1 inhibitors also have the potential to be effective cancer treatments. Inhibition of USP1 will lead to the destabilisation of CHK1 and other DNA repair pathway proteins, and, similar to CHK1i, result in the accumulation of damaged DNA, genomic catastrophe, and tumour cell death. In this paper, genetic deletion of the c-Rel NF-κB subunit leads to loss of USP1 and consequently CHK1 expression, resulting in inactivation of this pathway. However, loss of this pathway can be overcome by activation of compensatory bypass pathways (not shown), which is described in Hunter et al. [38]. Figure partially created using Biorender.

Loss of c-Rel has many effects in the Eµ-Myc lymphoma model, underlining the critical role this NF-κB subunit plays in this context [40]. Indeed, our data demonstrates that these Eµ-Myc/*cRel^−/−^* lymphoma cells undergo a comprehensive rewiring of their cell signalling pathways. Here we have explored the basis for the resistance of Eµ-Myc/*cRel^−/−^* lymphomas to CHK1 inhibition and revealed a pathway regulating the response to DNA replication stress in cancer (Figure 7).

One dramatic finding underpinning the development of resistance to the CCT244747 CHK1 inhibitor in this model is the loss of CHK1 protein itself, together with other components of the DNA replication stress response and cell cycle pathways. The mechanisms underlying this effect are complex but at the core of this is down-regulation of the mRNA and protein of the USP1 DUB in Eµ-Myc/*cRel^−/−^* cells. USP1 has been previously linked to numerous components of the DNA damage response [27,28,55] and implicated as a CHK1 DUB [49]. We propose that c-Rel directly, or indirectly, regulates the expression of USP1. Consequently, in the absence of c-Rel, loss of USP1 results in destabilisation of CHK1 and other pathway components. Contributing towards this effect, is the parallel loss of Claspin expression [19]. Whether it is the initial loss of Claspin, thus breaking the link between ATR and CHK1, that leads to subsequent effects via USP1 is not known. Nonetheless, it is probable that the parallel loss of USP1 and Claspin works synergistically to down-regulate CHK1 protein levels and activity. This complexity, however, ultimately creates a simple explanation for CHK1 inhibitor resistance in the Eµ-Myc/*cRel^−/−^* lymphoma cells: the target of the drug is no longer present (Figure 7).

Several USP family members are being exploited as potential targets as anti-cancer agents. For example, there are a number of compounds targeting USP7, the DUB known to target c-Myc as well as other proteins [56], in clinical development. Interestingly, USP7 inhibition sensitises AML cells to the chemotherapeutic agent cytarabine by destabilising CHK1 protein [57]. USP1 inhibitors are also now of interest given the role of USP1 in controlling multiple DNA damage response (DDR) pathways [27,28,55], and the reported overexpression of USP1 in certain tumour types (including sarcomas (reviewed in [29])), suggesting that inhibition of USP1 will remove a key node controlling various points of the DDR, leading to genomic catastrophe and cancer cell death. In fact, commercially available USP1 inhibitors have shown efficacy in models of prostate, breast and colorectal cancer [53,58,59]. Here, given our data and a previous report [49] that USP1 acts as a CHK1 DUB, we demonstrated that inhibition of USP1 using ML323 effectively killed wild-type Eµ-Myc lymphoma cells (Figure 6E). These tumours rely on ATR, CHK1 and also USP1 activity for their survival, suggesting that targeting USP1 would be an alternative strategy for treating cancers with high levels of MYC-induced replication stress (Figure 7). ML323 and other commercially available inhibitors of the USP1/UAF-1 complex act by targeting the DUB complex as opposed to the USP1 active site [52]. ML323 exhibited good selectivity over the other USP family members its activity was assayed against [60]. However, more potent and specific inhibitors are required for future clinical use.

Recently, there have been other reports of cross-talk between the replication stress pathway and USP1. USP1 was found to be up-regulated in BRCA1 mutant tumours where it appears to stabilise and protect the replication fork, thereby promoting survival in these cells with on-going DNA damage due to BRCA1 loss or mutation [55]. Interestingly, there is also a report suggesting that ATR and ATM can directly phosphorylate USP1 following treatment with the chemotherapeutic agent, cisplatin. Once phosphorylated, USP1 binds to and deubiquitinates Snail, resulting in resistance to cisplatin and an increased metastatic potential [61]. These studies highlight further levels of cross-talk between the USP1 and the replication stress response. In this context, it is also interesting to consider other data from our lab where we have demonstrated that phosphorylation of the RelA subunit on Thr 505 induces a pro-apoptotic form of NF-κB in response to cisplatin [9–11]. We have also shown that mutation of T505 RelA to alanine results in the earlier onset of cancer [17,19], alongside an enhanced invasive phenotype [9,19], and resistance to CHK1 inhibitors [19]. Figure 5B,C and Supplementary Figure S6B show a reduction in USP1 also occurs in the Eµ-Myc/*RelA^T505A^* lymphoma cells, and this in part may also contribute to the resistance to CHK1 inhibitors we have previously observed in these cells [19]. However, we do not observe loss of CHK1 protein in the Eµ-Myc/*RelA^T505A^* lymphomas. Unlike the Eµ-Myc/*cRel^−/−^* lymphoma cells (Figure 3A), we do not find CHK1 transcript levels to be down-regulated in Eµ-Myc/*RelA^T505A^* lymphomas (Supplementary Data File S5). This suggests that in the absence of a parallel transcriptional effect, the levels of USP1 remaining in Eµ-Myc/*RelA^T505A^* are sufficient to maintain CHK1 stability and any contribution of USP1 towards to phenotype of these lymphomas would come from effects on other target proteins [19].

Previous CHK1 inhibitor studies have shown other potential routes of drug sensitivity and resistance. An analysis of MK-8776 (performed in multiple sensitive and resistant cell lines) revealed up-regulation of CDK2 and Cyclin A in responsive cells after treatment with this CHK1 inhibitor, with an associated increase in double stranded DNA breaks [62]. Drug sensitivity was also associated with accumulation of CDC25A [62]. In contrast, cells that were MK-8776 resistant failed to dephosphorylate and thus activate CDK2 [62]. Another study found that cells deficient in MRE11 were resistant to MK-8776 mediated CHK1 inhibition [63]. Investigation of the CHK1 inhibitor LY2603618 (Rabusertib) in head and neck cancer cell lines also found that sensitivity to the drug was dependent on CDK activity, reporting that elevated CDK1 levels were indicative of reduced drug sensitivity, potentially due to up-regulation of origin firing and thus the ability to overcome S phase replication stalling [64]. Research into the PF-00477736 CHK1 inhibitor resistant mantle cell lymphoma cell line JEKO-1, showed that resistant cells had a shorter S phase and a reduced expression of cell cycle checkpoint proteins, including cyclin D1 [65]. These studies all contrast with our analysis, where we found that Eµ-Myc/*cRel^−/−^* lymphomas exhibited reduced levels of CDK1 and CDK2. It is therefore likely that the mechanism used in the development of CHK1 inhibitor resistance will be dependent on the tumour context, with both the cell type and oncogene/tumour suppressor status having a key role. Nonetheless, our data suggests that down-regulation or mutation of USP1 is likely to be a common feature arising in patients undergoing therapy involving a CHK1 inhibitor.

One caveat of this study is that it does not address how relevant this data is to human cancer in general and the use of CHK1 inhibitors clinically. However, key components of the work from Eµ-Myc lymphomas, such as down-regulation of CHK1 and USP1 protein, were also seen in independently derived populations of CCT244747 resistant U2OS cells, and SRA-737 resistant Huh-7 cells (Figure 5). Moreover, down-regulation of USP1 in WT U2OS cells resulted in loss of CHK1 protein and CCT244747 resistance. Nonetheless, our data suggests that USP1 is a novel target for MYC-driven tumours and that this warrants further investigation.

## Methods

### Ethics statement

All mouse experiments were approved by Newcastle University's Animal Welfare and Ethical Review Board. All procedures, including the of breeding genetically modified mice, were carried out under project and personal licenses approved by the Secretary of State for the Home Office, under the United Kingdom's 1986 Animal (Scientific Procedures). Animals were bred in the Comparative Biology Centre, Newcastle University animal unit, according to the FELASA Guidelines.

### Mouse models

*c-Rel^−/−^* mice were provided by Dr Fiona Oakley (Newcastle University), *RelA*^T505A^ knock in mice were generated by Taconic Artemis (Germany) using C57Bl/6 ES cells [17] and Eµ-Myc mice were purchased from The Jackson Laboratory, Maine, U.S.A.. C57Bl/6 mice used for re-implantation studies were purchased from Charles River (U.K.). Male Eµ-Myc transgenic mice that were used as breeding stock were omitted from the survival analysis. In all experiments, the relevant pure C57Bl/6 (WT) strain was used as a control. No blinding of groups in mouse studies was performed. All mice were designated to an experimental group dependent on their genotype.

### Drugs and compounds

CCT244747 was synthesised as described [67] by MedKoo Biosciences. SRA-737 was purchased from Selleckchem (S8253). CHK2 inhibitor CCT241533 was purchased from Tocris. All other compounds were purchased from Sigma–Aldrich.

### Resistant cell line generation

U2OS cells were cultured in increasing concentrations of CCT24474, starting with the IC_50_ concentration of 1 µM. The concentration of CCT244747 was doubled at each passage to a final concentration of 8 µM. WT controls were given an equivalent amount of DMSO to account for any DMSO-related toxicity.

Huh-7 cells were cultured in increasing concentrations of SRA-737 [19] starting with the IC_50_ concentration of 1 µM. The concentration of SRA-737 was doubled at each passage to a final concentration of 16 µM.

### siRNA knockdown transfections

U2OS cells were transfected with 10 nM siRNA targeting either USP1 (L-006061-00), USP14 (L-006065-00) or cRel (L-004768-00) (ON-TARGET plus Smart pool, Dharmacon) or a Non-specific siRNA control (D-001810-00) using Dharmafect 4 transfection reagent (T-2004-03), according to manufacturer's protocols. Cells were harvested, or used in downstream assays 72 h post-transfection, once target depletion had been confirmed.

### Cell viability assays

Freshly isolated Eµ-Myc, Eµ-Myc/*cRel^−/−^* or Eµ-Myc RelA^T505A^ tumour cells (5 × 10^5^ per well), or WT or CHK1i resistant U2OS (5 × 10^3^ per well) were seeded into 96-well plates. Increasing concentrations of CHK1 inhibitor, CCT244747, or solvent controls were added to three replicate wells. After 96 h, viability was quantified using the PrestoBlue Cell Viability Reagent (A13262, ThermoFisher Scientific, U.K.), according to manufacturer's instructions.

### Cell survival assays

Exponentially growing WT or CHK1i resistant U2OS were treated for 24 h with 1 µM CHK1 inhibitor, CCT244747, 30 µM USP1 inhibitor, ML323 or solvent controls before re-seeding onto Petri dishes at known cell number (750, 1000, 1500, 2500 or 5000 cells/dish). Colonies were fixed 21 days later with methanol:acetic acid (3 : 1) and stained with 0.4% (w/v) Crystal Violet. Cloning efficiencies were normalised to untreated controls.

### Gene expression analysis using quantitative real-time PCR

Total RNA was puriﬁed from snap frozen Eµ-Myc, Eµ-Myc/*cRel^−/−^* or or Eµ-Myc RelA^T505A^ tumour samples by homogenisation using Precellys 24 ceramic mix bead tubes (431-0170, Stretton Scientific Ltd) in a Precellys 24 benchtop homogeniser (Stretton Scientific Ltd) at 6500 rpm for 30 s. Following this, samples were passed through Qiashredders (79656, Qiagen, Crawley, U.K.) and RNA was purified using the Qiagen RNeasy mini kit (74004) according to manufacturer's instructions. Total RNA from exponentially growing WT or CHK1i resistant U2OS was extracted using the PeqGold total RNA extraction kit (Peqlab), according to manufacturer's instructions.

RNA was measured for purity and concentration with the NanoDrop1000 (ThermoFisher Scientific) and reverse transcribed using the Quantitect Reverse transcription Kit (Qiagen) according to manufacturer's instructions. Quantitative real-time PCR was performed on 20 ng cDNA, in triplicate, using predesigned Quanititect Primer assays (Qiagen) to the following murine genes; *Clspn* (QT00154609)*, Chek1* (QT00109179)*, Usp1* (QT00177352)*, Usp14* (QT00171577) and human genes *CLSPN (*QT00027804)*, CHEK1(*QT00006734)*, USP1* (QT00008568) and *USP14 (*QT00063182*).* These samples were run and analysed on a Rotor-gene Q system (Qiagen), using murine *Rpl13a* (QT00267197) or human *RPL13A* primers as an internal control. All CT values were normalised to *Rpl13a*/*RPL13A* levels.

### Western blotting

Whole cell extracts were prepared from snap frozen pieces of Eµ-Myc, Eµ-Myc/*cRel^−/−^* or Eµ-Myc RelA^T505A^ tumour tissue. Snap frozen tumour was lysed in PhosphoSafe™ Extraction Reagent (71296, Merck Millipore) using the Precellys24 ceramic mix bead tubes (Stretton Scientific Ltd) in a Precellys®24 homogeniser (Stretton Scientific Ltd) at 6500 rpm for 30 s, then extracted according to the PhosphoSafe™ Extraction Reagent manufacturer's instructions. In the case of cell lines samples, cell pellets were washed with ice-cold PBS, and lysed using PhosphoSafe™ Extraction Reagent (Merck-Millipore, Watford, U.K.), according to manufacturer's protocols. Protein quantification was undertaken using the BCA protein assay, and samples resolved by standard denaturing SDS–PAGE gels using the Criterion Gel System (3450034, Bio-Rad). Samples were transferred onto PVDF membrane (GVWP04700, Merck-Millipore) before being probed with the primary antibody. Horseradish peroxidase-conjugated secondary antibodies and enhanced chemiluminscence reagent (32106, Thermo-scientific, U.K.) were used for detection.

### Antibodies

Antibodies used were CHK1 (phospho S345) (2341 Cell Signaling), CHK1 (2360 Cell Signaling), USP1 (14346-1-AP Proteintech), USP14 (14517-1-AP Proteintech), γH2AX (2577 Cell Signaling), CDC25B (9525 Cell Signaling), CHK2 (3440 Cell Signaling), Karyopherin (sc-137016 Santa Cruz), CDK2 (2546 Cell Signaling), CDK1 (phospho Y15) (4539 Cell Signaling), RPA2/RPA32 Ser 33 (10148 Cell Signaling), RPA2/RPA32 (52488 Cell Signaling), ATRIP (11327-1-AP Proteintech), Rad17 (13358-1-AP Proteintech), ATR Ser 428 (2853 Cell Signaling), ATR (13934 Cell Signaling) and CDK1 (9116 Cell Signaling). Antibodies to the murine form of Claspin was generated by Moravian Biotechnologies. Anti-rabbit IgG (A6154 Sigma and 7074 Cell Signaling) and anti-mouse IgG (7076 Cell Signaling) HRP-linked secondary antibodies were used for western blot detection.

### Eµ-Myc mice studies

Eµ-Myc/*cRel^−/−^* offspring were generated by mating *c-Rel^−/−^* female mice with Eµ-Myc male mice, further Eµ-Myc*/c-Rel^−/−^* mice were generated by crossing Eµ-Myc*/c-Rel^+/−^*males with *c-Rel^−/−^* female mice [40]. Eµ-Myc*/RelA*^T505A*+/−*^ offspring were generated by mating *T505A* female mice with Eµ-Myc male mice, further Eµ-Myc/*RelA*^T505A^ mice were generated by crossing Eµ-Myc*/T505A^+/−^* males with *T505A* female mice [19]. Eµ-Myc transgenic mice, and the associated crosses were monitored daily and were killed at pre-determined end-points, defined as the animal becoming moribund, losing bodyweight/condition and/or having palpable tumour burden at any lymphoid organ site. Moribund mice were necropsied and single cell suspensions were prepared from tumour-bearing organs. Mice were humanely killed by cervical dislocation. No anaesthesia was used at any point during any studies described. Briefly, lymph nodes, spleen or thymus were homogenised through a cell strainer, and single cell suspension collected in DMEM (Lonza) supplemented with 10% FBS, 5 mM l-glutamine, 5 mM sodium pyruvate, 1 µM l-asparagine and 50 µM β-mercaptoethanol (Sigma–Aldrich). These cell suspensions were then frozen in 90% FBS/10% DMSO for long-term storage and transplantation.

### Re-implantation studies

For tumour therapy studies, 2 × 10^6^ Eµ-Myc or Eµ-Myc/*cRel^−/−^* tumour cells from male mice were transplanted intravenously (IV) via the lateral tail vein into 8-week old male C57BL/6 recipients. Mice were monitored daily using parameters such as their bodyweight and food and water consumption to assess disease progression. Mice were necropsied when they became moribund and the tumour burden assessed.

Oral administration of the CHK1 inhibitor, CCT244747, prepared as previously described [41], or vehicle control (65% PEG-400, 20% Tween-20, 10% H_2_O, 5% DMSO (all Sigma–Aldrich)) was initiated when tumours became palpable (∼10 days after inoculation of Eµ-Myc cells, and 20 days after inoculation of Eµ-Myc*/c-Rel^−/−^* cells). During efficacy studies, CCT244747 was given as a single agent, bolus dose (100 mg/kg p.o.) for nine consecutive days. Lymphoid tumour burden and final tumour weights were measured at necropsy 24 h after the final dose. For acute proteomic studies, CCT244747 was given as a single agent, bolus dose (100 mg/kg p.o.) once ∼14 days after inoculation of Eµ-Myc cells and 25 days after inoculation of Eµ-Myc*/c-Rel^−/−^* cells, with the tumours being necropsied either 8, 24 or 48 h after dosing.

Intra-peritoneal administration of the USP1 inhibitor, ML323 (HY-17543), prepared as previously described [54,58], or vehicle control (2% carboxymethyl cellulose (419338, Sigma–Aldrich)) was initiated when tumours became palpable and given as a single agent, (10 mg/kg i.p) for nine consecutive days. Lymphoid tumour burden and final tumour weights were measured at necropsy 24 h after the final dose.

### Cycle cell analysis

Single cell suspensions from Eµ-Myc WT and Eµ-Myc/*cRel^−/−^*were permeabilised with ice-cold 70% ethanol whilst being vortexed, before a 30 min incubation on ice. Cells were pelleted and washed twice with PBS before staining with 20 µg/ml RNase A and 50 µg/ml propidium iodide (PI). Cells were incubated for 20 min in the dark before analysis on the FACSCanto II flow cytometer (BD Immunocytometry Systems, San Jose, CA, U.S.A.) equipped with a 488 nm (blue) laser. At least 25 000 events were collected and data were analysed post-acquisition with FlowJo software (v10, TreeStar). Doublets and debris were excluded using PI width vs PI area and remaining cells were plotted as a histogram using PI area vs count.

### Proteomics and analysis

Tissue extracts were prepared from snap frozen pieces of Eµ-Myc, or Eµ-Myc/c-Rel*^−/−^* splenic tumours. Briefly, tissue samples were suspended in 100 mM triethylammoniumbicarbonate (TEAB) with a mixture of protease and phosphatase inhibitors (cOmplete Mini EDTA-free protease inhibitor cocktail plus PhoSTOP phosphatase inhibitor cocktail, both obtained from Roche), homogenised by bead beater, and sonicated on ice. Lysed extracts were incubated with 0.1% (w/v) Rapigest SF (Waters) for 10 min at 80 °C, left to cool, and incubated for 10 min on ice with Benzonase endonuclease (Merck Millipore) to digest nucleic acids. Samples were centrifuged (14 000***g***, 10 min at 4°C) to pellet cell debris. Protein concentration of the clarified lysate was ascertained by Bradford assay. Protein (200 µg) from each sample was aliquoted for protein digestion.

Disulfide bonds were reduced (4 mM DTT in 100 mM TEAB, 10 min at 60°C) and free cysteines alkylated with iodoacetamide (14 mM in 100 mM TEAB, for 30 min, RT in the dark). Iodoacetamide was quenched by addition of DTT to a final concentration of 7 mM. Proteins were digested with 2% (w/w) trypsin overnight at 37°C with gentle agitation. Resultant peptides were labelled with TMT 6-plex reagents (Thermo Scientific) at an 8 : 1 tag:protein ratio as per the manufacturer's instructions, with labels assigned to samples randomly for the first biological replicate and shifted for each subsequent replicate. The labelling reaction was quenched by addition of 0.3% (v/v) hydroxylamine (Thermo Scientific) in 100 mM TEAB. TMT labelled peptides were mixed and dried to completion by vacuum centrifugation before re-suspending in 100 mM TEAB/ 1% TFA to hydrolyse the Rapigest SF (RT, 10 min). Insoluble Rapigest SF cleavage product was removed by centrifugation (13 000***g*** for 15 min at 4°C), and the sample desalted using C18 spin columns (Pierce, #89852) as per the manufacturers protocol, prior to strong cation exchange using stage tips (packed in-house with five disks per 200 µl tip as described previously [68] (Empore Supelco 47 mm Cation Exchange disk, #2251)). Each mixed labelled peptide sample was split across 8 tips, with peptides passed through the equilibrated stage tips twice. Bound peptides were eluted with 5% NH_4_OH (3 × 100 µl) and dried to completion using a vacuum centrifuge.

Peptides were fractionated using basic reverse-phase liquid chromatography as described [68], with 65 fractions collected, partially dried by vacuum centrifugation, and concatenated into five pools. For each pool, 5% was aliquoted and dried to completion prior to MS analysis. The remaining 95% was subjected to TiO_2_-based phosphopeptide enrichment, as described previously [69].

Total protein and phosphopeptide enriched fractions were analysed by LC–MS/MS using an UltiMate 3000 RSLCTM nano system (Dionex) coupled in-line with a Thermo Orbitrap Fusion Tribrid mass spectrometer (Thermo Scientific). Peptides were loaded onto the trapping column (PepMap100, C18, 300 µm × 5 mm, Thermo Scientific) using partial loop injection with 2% acetonitrile (ACN), 0.1% TFA at a flow rate of 9 µl/min for 7 min. Peptides were resolved on an analytical column (Easy-Spray C18, 75 µm × 500 mm, 2 µm bead diameter) using a gradient from 96.2% A (0.1% formic acid):3.8% B (80% ACN, 0.1% formic acid) to 50% B over either 120 min (single injection for phosphopeptide-enriched samples and two injections for total protein samples) or 240 min (single injection for total protein samples only) at a flow rate of 300 nl/min. Full MS1 spectra were acquired in the Orbitrap over *m/z* 375-2000 (60 K resolution at m/z 200), with a maximum injection time of 50 ms and an ACG target of 4 × 10^5^ ions. Data-dependent MS2 analysis was performed using a top speed approach (3 s cycle time) with peptides fragmented by collision-induced dissociation [70] at a normalised collision energy (NCE) of 35%, with fragment ions detected in the ion trap (maximum injection time of 50 ms, ACG target of 1 × 10^4^). Following acquisition of each MS2 spectrum, a synchronous precursor selection (SPS) MS3 scan was performed on the top 10 most intense fragment ions, with SPS-MS3 precursors fragmented using higher energy collision-induced dissociation (HCD), at an NCE of 65%, and analysed using the Orbitrap over *m/z* 100–500 (50 K resolution at m/z 200) with a maximum injection time of 105 ms and an ACG target of 1e5 [71,72].

Analysis of MS data, with quantification of TMT reporter ion distributions, was performed using Proteome Discoverer 2.4 (PD 2.4) in conjunction with MASCOT (v2.6) and Percolator. For peptide identification from MS2 spectra, raw data files were converted to mzML format and searched in MASCOT against the Mouse UniProt reviewed database (Downloaded 25/04/2018; 16,966 sequences) with parameters set as follows: MS1 tolerance of 10 ppm; MS2 tolerance of 0.6 Da; enzyme specificity was set as trypsin with two missed cleavages allowed; carbamidomethylation of cysteine and TMT 6-plex modifications (on peptide N-termini and lysine side chains) were set as fixed modifications; oxidation of methionine and acetylation of protein N-termini were set as variable modifications, with the addition of phosphorylation (at serine, threonine or tyrosine residues) for phosphopeptide-enriched samples. Percolator was used for control of false discovery rates with a target FDR of 0.05. For phosphopeptide-enriched samples, the ptmRS node, operated in phosphoRS mode, was added to the PD 2.4 workflow for phosphosite localisation. In parallel with peptide identification, relative quantification of TMT 6-plex reporter ions was performed in PD 2.4 using the ‘Reporter ions quantifier’ node, to quantify reporter ions from MS3 spectra with a peak integration tolerance of 20 ppm using the ‘most confident centroid’ integration method. Normalisation to total peptide amount was performed within PD 2.4, with peptide group abundances summed for each sample and a normalisation factor calculated from the sum of each sample and the maximum sum in all files.

Quantitative ratios were calculated for each biological replicate to look for protein/phosphopeptide changes. Quantitative ratios were log_2_ transformed and, for all proteins/phosphopeptides quantified in at least three out of five bioreps, statistical analysis was performed in R using the LIMMA package, using a *P* ≤ 0.05 significance cut off. Pearson correlation analysis was performed in R using the ggscatter package, with a linear regression line and 95% confidence intervals included on each plot. The mass spectrometry proteomics data have been deposited to the ProteomeXchange Consortium (http://proteomecentral.proteomexchange.org) via the PRIDE partner repository [66] with the dataset identifiers Project accession: PXD026203 & Project DOI: 10.6019/PXD026203.

Please note that data from control samples from WT Eµ-Myc mice is also used in the analysis of changes in RelA T505A Eµ-Myc lymphomas described elsewhere [19]. Consequently, this description of the methods is duplicated in that paper. Moreover, some figures using these control samples are also duplicated in that study. These are clearly indicated in figure legends. We have compiled Supplementary Data from proteomics analysis in the study into a single file (Supplementary Data File S1), which is also attached to the other papers that analyse this data [19,38].

### RNA-Seq and analysis

RNA was extracted as described above and sample quality analysed using Tapestation automated electrophoresis (Aglient) according to manufacturer's instructions. Sample RNA Integrity Number (RIN) score exceeded six in all cases. mRNA-Seq libraries were prepared using the Illumina TruSeq Stranded mRNA kit following manufacturer's reference guide and sequenced on an Illumina NextSeq 500 high-output 75 cycle flow cell, generating 25 million 75 bp single reads per sample. The raw sequence data quality was first inspected using FastQC and MultiQC. Transcript counts were generated via Salmon [73] using Release M20 (GRCm38.p6) of the mouse genome (for the mouse samples) and Release 31 (GRCh38.p12) of the human genome (for the human samples).

The quantification files were imported into R for gene-level analyses using tximport [74] and the differential gene expression analyses were carried out using DESeq2 [75]. The data has been deposited on ENA (https://www.ebi.ac.uk/ena/browser/home) with the accession number PRJEB45284.

Please note that data from control samples from WT Eµ-Myc mice is also used in the analysis of changes in RelAT505A Eµ-Myc lymphomas described elsewhere [19]. Data from the T505A Eµ-Myc mice is also analysed in our analysis of bypass pathways [38]. Consequently, this description of the methods is duplicated in these papers. Moreover, some figures using these control samples are also duplicated in that study. These are clearly indicated in figure legends. We have compiled Supplementary Data from RNA Seq analysis in the study into single files (Supplementary Data Files S5–8), which, for the Eµ-Myc mouse data, are also attached to the other papers that analyse this data [19,38].

### STRING, Venn diagram and David analysis

STRING analysis was performed using version 11.0 at https://string-db.org/ [76]. Where indicated CHEK1 was manually added to the protein list to determine connections to phosphoproteins identified from the proteomics analysis. Analysis of connections was performed under with medium or high confidence as described in figure legends, using homo sapiens as the species setting. Connections were limited to query proteins only. In all STRING analysis shown, the lines connecting proteins indicate both functional and physical associations with the line thickness indicates the strength of data support. Details on proteins analysed and connections are in Supplementary Data File S2. Venn diagram analysis was performed at http://bioinformatics.psb.ugent.be/webtools/Venn/ with figures being created using https://www.biovenn.nl/index.php. See Supplementary Data File S3 for more details. Functional annotation clustering was performed using https://david.ncifcrf.gov/home.jsp.

### Statistical analysis

GraphPad Prism software (http://www.graphpad.com, V6.0) was used for statistical analysis. Except where stated in figure legends, unpaired *t*-tests or One-way ANOVA were used to calculate *P*-values (*P*-values of *P* < 0.05 were considered signiﬁcant). Pearson correlations were performed using the ggscatter package in R, with linear regression lines fitted with 95% confidence intervals.

## Data Availability

The mass spectrometry proteomics data have been deposited to the ProteomeXchange Consortium (http://proteomecentral.proteomexchange.org) via the PRIDE partner repository [66] with the dataset identifiers Project accession: PXD026203 & Project DOI: 10.6019/PXD026203. RNASeq data has been deposited on ENA (https://www.ebi.ac.uk/ena/submit/sra/#home) with the accession number PRJEB45284. The authors are happy to provide all original data, and for this to be shared on Figshare as appropriate.

## Open Access

Open access for this article was enabled by the participation of University of Liverpool in an all-inclusive *Read & Publish* agreement with Portland Press and the Biochemical Society under a transformative agreement with JISC.

## Abbreviations

ACN: acetonitrile
AML: acute myeloid leukaemia
ATM: ataxia telangiectasia mutated
ATR: ataxia telangiectasia and Rad3 related
DDR: DNA damage response
DUBs: Deubiquitinases
FDR: false discovery rate
NCE: normalised collision energy
PI: propidium iodide
RPA: Replication Protein A
SPS: synchronous precursor selection
TEAB: triethylammoniumbicarbonate
TMT: tandem mass tag
USP1: Ubiquitin-specific protease 1
