# Supplementary material for: Regulation of CHK1 inhibitor resistance by a c-Rel and USP1 dependent pathway
Source: Biochem J. 2022 Oct 14;479(19):2063–86. doi: 10.1042/BCJ20220102 (PMC9704646; doi:10.1042/BCJ20220102)

Supplementary Information

Regulation of CHK1 inhibitor resistance by a c-Rel and USP1 dependent pathway

Jill E. Hunter[1], Amy E. Campbell[2], Nicola L. Hannaway[1], Scott Kerridge[1], Saimir Luli[3], Jacqueline A. Butterworth[1], Helene Sellier[1], Reshmi Mukherjee[1], Nikita Dhillion[1], Praveen Dhondurao Sudhindar[1], Ruchi Shukla[1], Philip J. Brownridge[2], Hayden L. Bell, Jonathan Coxhead[1], Leigh Taylor[1], Peter Leary[4], Megan S.R. Hasoon[4] Ian Collins[5], Michelle D. Garrett[6], Claire E. Eyers[2*] and Neil D. Perkins[1*]

[1] Newcastle University Biosciences Institute

Faculty of Medical Sciences

Newcastle University

Newcastle Upon Tyne, NE2 4HH, UK

[2]Centre for Proteome Research, Department of Biochemistry and Systems Biology,

Institute of Systems, Molecular and Integrative Biology,

University of Liverpool,

Liverpool L69 7ZB, U.K.

[3]Newcastle University Clinical and Translational Research Institute

Preclinical In Vivo Imaging (PIVI)

Faculty of Medical Sciences

Newcastle University

Newcastle Upon Tyne, NE2 4HH, UK

[4]Bioinformatics Support Unit,

Faculty of Medical Sciences

Newcastle University

Newcastle Upon Tyne, NE2 4HH, UK

[5]The Institute of Cancer Research

Sutton, SM2 5NG, UK

[6]School of Biosciences,

Stacey Building,

University of Kent,

Canterbury, Kent, CT2 7NJ, UK

* joint corresponding author

Tel. 0191 2082245

Fax. 0191 2087424

Email: neil.perkins@ncl.ac.uk

Email: Claire.Eyers@liverpool.ac.uk

Supplementary Figures

Supp Figure 1

(A) Line graphs showing the mean response of the five reimplanted Eμ-Myc and Eμ-Myc/*cRel-/-* (blue) tumours and their response to CCT244747 in further lymphoid organs. Each of the 5 tumours was implanted into 6 syngeneic recipient C57Bl/6 mice, 3 were treated with CCT244747 (100 mg/kg p.o), and 3 with vehicle control, for 9 days once tumours became palpable. A response was defined as a significant reduction (or increase) in tumour burden ($P<0.05$) using unpaired Student's t-tests. Please note that the data from WT Eμ-Myc mice shown here is also used in our study on RelA T505A Eμ-Myc lymphomas [19]. These experiments were performed in parallel as part of the same larger study.

(B) Eμ-Myc, Eμ-Myc/*cRel-/- and* Eμ-Myc/*T505A* tumours were treated with 0.5 μM or 1 μM CCT244747 (or vehicle control) for 96 hours *ex vivo*. Eμ-Myc tumour cells show a reduced cell viability compared with the Eμ-Myc/*cRel-/-* or Eμ-Myc/*T505A* tumour cells at all doses of CCT244747 tested.

Supp Figure 2

(A) Schematic illustrating the workflow for proteomics experiments. Splenic tumours from Eμ-Myc or Eμ-Myc/*cRel-/-* mice were necropsied 8 hours post-treatment with either CHK1 inhibitor CCT244747 or vehicle control. Proteins were extracted and digested with trypsin prior to peptide labelling with tandem mass tags (TMT). Differentially labelled peptides from each treatment condition were mixed then fractionated via basic reverse-phase liquid chromatography, initially into 65 fractions which were concatenated into 5 pools. For each pool, 5% of the material was analysed by LC-MS/MS to obtain relative-quantification of total protein levels whilst the remaining 95% was subject to titanium dioxide (TiO_2)-based phosphopeptide enrichment prior to LC-MS/MS analysis for phosphoproteomic analysis.

(B & C) Volcano plots demonstrating a significant number of CCT244747 effects in Eμ-Myc WT lymphomas on both the total (A) and phospho (B) proteome, with both down- (red dots) and up-regulation (blue dots) being observed.

Please note that figures (B) and (C) showing data from WT Eμ-Myc lymphomas are replicated in another manuscript [19], where they are used for comparison with data from RelA T505A Eμ-Myc lymphomas.

Supp Figure 3

(A) Table detailing the number of phosphopeptides (orange column) and proteins (blue column) that were significantly different when the genotypes and/or treatment arms were compared after proteomic analysis in the acute CCT244747 study. A significant different was defined as an adjusted p value of <0.05

(B) Venn diagram illustrating that there is a high number of phosphosite changes in the Eμ-Myc/*cRel-/-* lymphomas without inhibitor treatment, but there is also a high level of overlap between these and the WT lymphomas following CCT244747 treatment.

(C) Venn diagram illustrating that there is a high number of total protein changes in the Eμ-Myc/*cRel-/-* lymphomas without inhibitor treatment, but there is also a high level of overlap between these and the WT lymphomas following CCT244747 treatment.

(D) Wider STRING analysis of the proteins associated with the 589 down-regulated phospho-peptides in the Eμ-Myc/*cRel-/-* lymphomas. Analysis performed under medium confidence setting as in Fig 2D but CHK1 was not added manually to the protein list. The red box indicates the same cluster of proteins with known linkages to CHK1 or CHK1 signalling, shown in Fig 2D. Positions of proteins within the network can change when CHK1 is added or removed but the connections themselves are not altered. See Supp Data File 2 for further details in linkages and analysis.

(E & F) String analysis performed as in Fig 2D and S3D but using the high confidence setting. In (E), to illustrate the links to CHK1, this was added manually into the analysis (circled in red), while (F) shows the same analysis without CHK1. Since the string analysis was limited to only the query proteins, this does not increase the number of connections apart from those to CHK1 itself (Supp Data File 2). Positions of proteins within the network can change when CHK1 is

added or removed but the connections themselves are not altered. See Supp Data File 2 for further details in linkages and analysis.

Supp Figure 4

Pearson correlation between fold changes (log_2) for proteins/phosphopeptides down-regulated in Eμ-Myc/c-Rel-/- lymphomas, and fold changes (log_2) for the same proteins/phosphopeptides in WT Eμ-Myc lymphomas after CCT244747 treatment, with both normalised to control treated WT Eμ-Myc lymphomas. Although there is a positive correlation in the changes observed at both protein and phosphopeptide level under these conditions, the magnitude of the fold change seen in WT Eμ-Myc lymphomas after CCT244747 treatment is generally much less than that observed in Eμ-Myc/c-Rel-/- lymphomas. Blue dots indicated proteins or phosphopeptides that are downregulated in both conditions. Black dots indicate phosphopeptides that are downregulated in Eμ-Myc/c-Rel-/- lymphomas but upregulated in WT Eμ-Myc lymphomas after CCT244747 treatment. The solid black line represents the linear regression line with the shaded region showing a 95% confidence interval. The dashed line shows where the regression line would fall if fold changes were identical between the compared conditions.

Supp Figure 5

(A) Table showing the log2 fold change (FC) and adjusted p value (pAdj) RNA Seq data for the 32 genes associated with the 'Activation of ATR in response to replication stress' (https://reactome.org/content/detail/R-HSA-176187) in the Eμ-Myc c-Rel$^{-/-}$ tumours when compared with Eμ-Myc WT tumours.

(B) Venn diagram showing the overlap between downregulated protein encoding transcripts (from RNA Seq data) vs down regulated proteins (from total proteome data) in REL-/- vs WT Eμ-Myc lymphomas.

(C) Western blot analysis of phospho-Ser345 CHK1, CHK1, CDK2 or ACTIN in snap frozen tumour extracts prepared from different re-implanted Eμ-Myc and Eμ-Myc/*cRel-/-* tumours to

those used in Fig 2B. Mouse inguinal lymph nodes were extracted 8 hours following a single dose of CCT244747. The expression of CHK1 and related pathway components are lost in Eμ-Myc/*c-rel-/-* tumours.

(D) Cycle cell analysis of Eμ-Myc WT and Eμ-Myc/*cRel-/-* lymphoma cells. Single cell suspensions from 6 different Eμ-Myc WT and Eμ-Myc/*cRel-/-* lymphomas were permeabilised and stained with propidium iodide (PI) before analysis by flow cytometry.

Supp Figure 6

(A) Western blot analysis of yH2AX or ACTIN in extracts prepared from WT and CCT244747 resistant U20S cells treated with 1 μM CHK1 inhibitor, CCT244747, 1 μM CHK2 inhibitor, CCT241533 or solvent controls.

(B) Western blot analysis of USP1, USP14 or ACTIN in snap frozen tumour extracts prepared from an additional re-implanted Eμ-Myc and Eμ-Myc/*cRel-/-* tumour. Mouse inguinal lymph nodes were harvested 8 hours following a single dose of CCT244747. USP1 and USP14 expression is lost in Eμ-Myc/*c-Rel-/-* tumours. Please note that the Actin blot from this figure is also used in another study (Fig. S2C lower panel) [19], where the same membrane was probed with antibodies to other proteins.

(C) CCT244747 resistant (CR) U20S cell lines are resistant to treatment with the USP1 inhibitor, ML323. Bar graph data showing clonogenic survival in WT and CR U20S following either treatment with 30 μM ML323 or solvent controls for 24 hours. Data was analysed using One-way ANOVA with multiple comparisons and Sidak's post-hoc test. P values of $p<0.05$ were considered significant.

(D) Further western blot analysis of independent isolates of WT or CCT244747 resistant (CR) U2OS cells treated with CCT244747, the USP1 inhibitor, ML323, or the Proteasome inhibitor MG-132, alone or in combination. Blots were probed for CHK1, USP1, yH2AX or ACTIN. Inhibition of USP1 in WT U2OS results in the loss of CHK1. Proteasomal inhibition in the CCT244747 resistant U2OS cells results in the stabilization of CHK1 protein.

Supplementary data files

Supp Data File 1 Proteomics.xlsx

Data from proteomics analysis of reimplanted Eμ-Myc lymphoma cells with either vehicle of CHK1i (CCT244747) treatment for 8 hours. Please note, this data file also accompanies two other manuscripts where we use Eμ-Myc lymphoma cells [19, 38].

Supp Data File 2 STRING interactions.xlsx

STRING interaction data analysing the links between phosphorylated proteins identified from phospho proteomics analysis.

Supp Data File 3 Venn diagrams.xlsx

Data files from Venn analysis of Eμ-Myc lymphoma cell proteomics and RNA Seq data

Supp Data File 4. Down regulated phosphopeptide analysis

Data analysing the phopshopeptides and phosphosites downregulated in Eμ-Myc/cRel-/- lymphomas or in Eμ-Myc WT lymphomas after CCT244747 treatment.

Supp Data File 5 RNASeq_all_genes_list_EuMyc.xlsx

Gene lists from RNA Seq analysis of reimplanted Eμ-Myc lymphoma cells with either vehicle of CHK1i (CCT244747) treatment for 8 hours. Please note, this data file also accompanies two other manuscripts where we use Eμ-Myc lymphoma cells [19, 38].

Supp Data File 6 RNASeq_counts_tximport_EuMyc.xlsx

Data for all genes and samples from RNA Seq analysis of reimplanted Eμ-Myc lymphoma cells. Please note, this data file also accompanies two other manuscripts where we use Eμ-Myc lymphoma cells [19, 38].

Supp Data File 7 RNASeq all_genes_list_U2OS.xlsx

Gene lists from RNA Seq analysis of wild type control and CHK1i (CCT244747) resistant U2OS cells with or without CCT244747 treatment for 24 hours.

Supp Data File 8 RNA Seq counts_tximport_U2OS.xlsx

Data for all genes and samples from RNA Seq analysis of U2OS cells.

Hunter et al., Figure S1

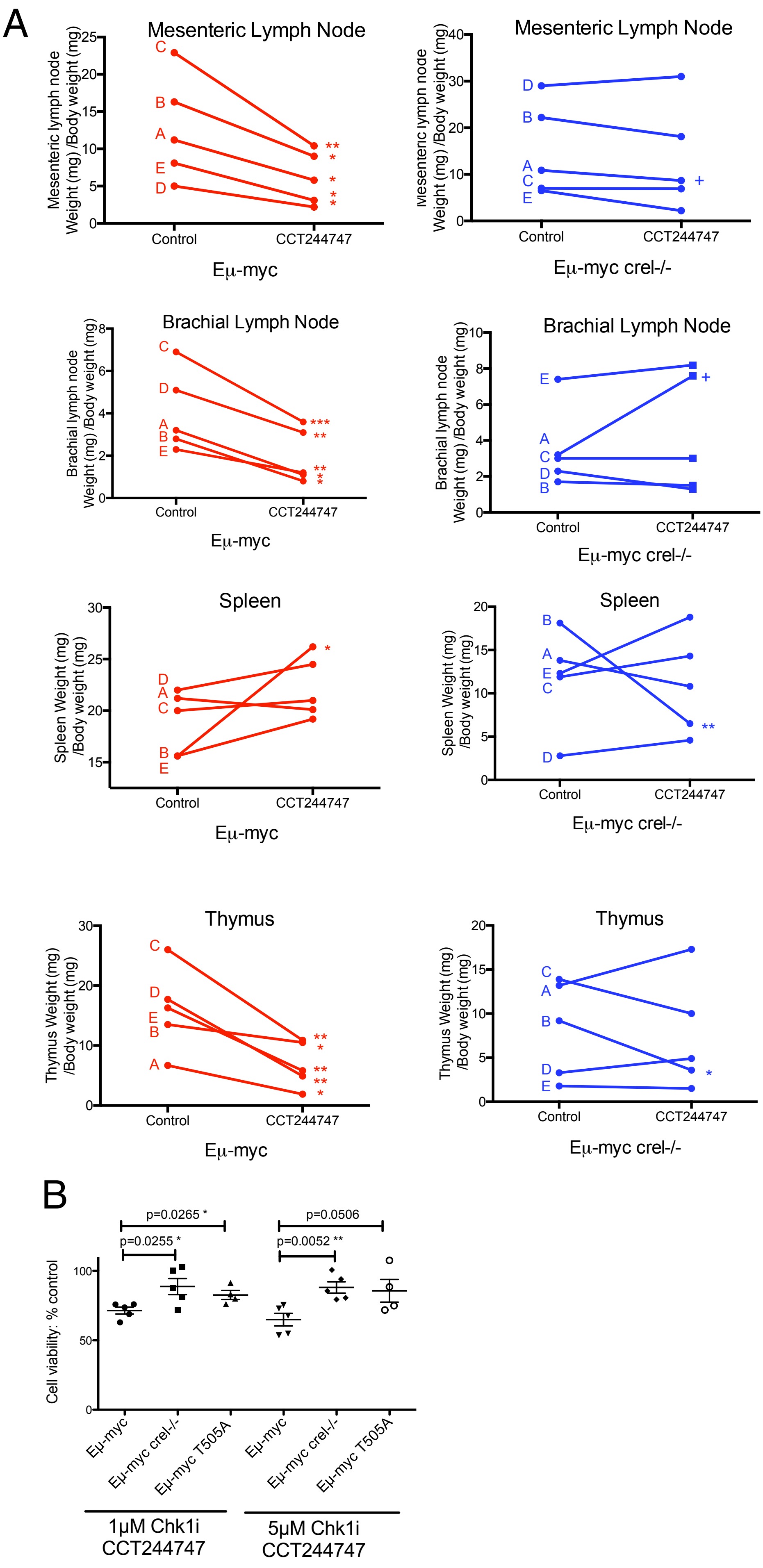

Hunter et al., Figure S2

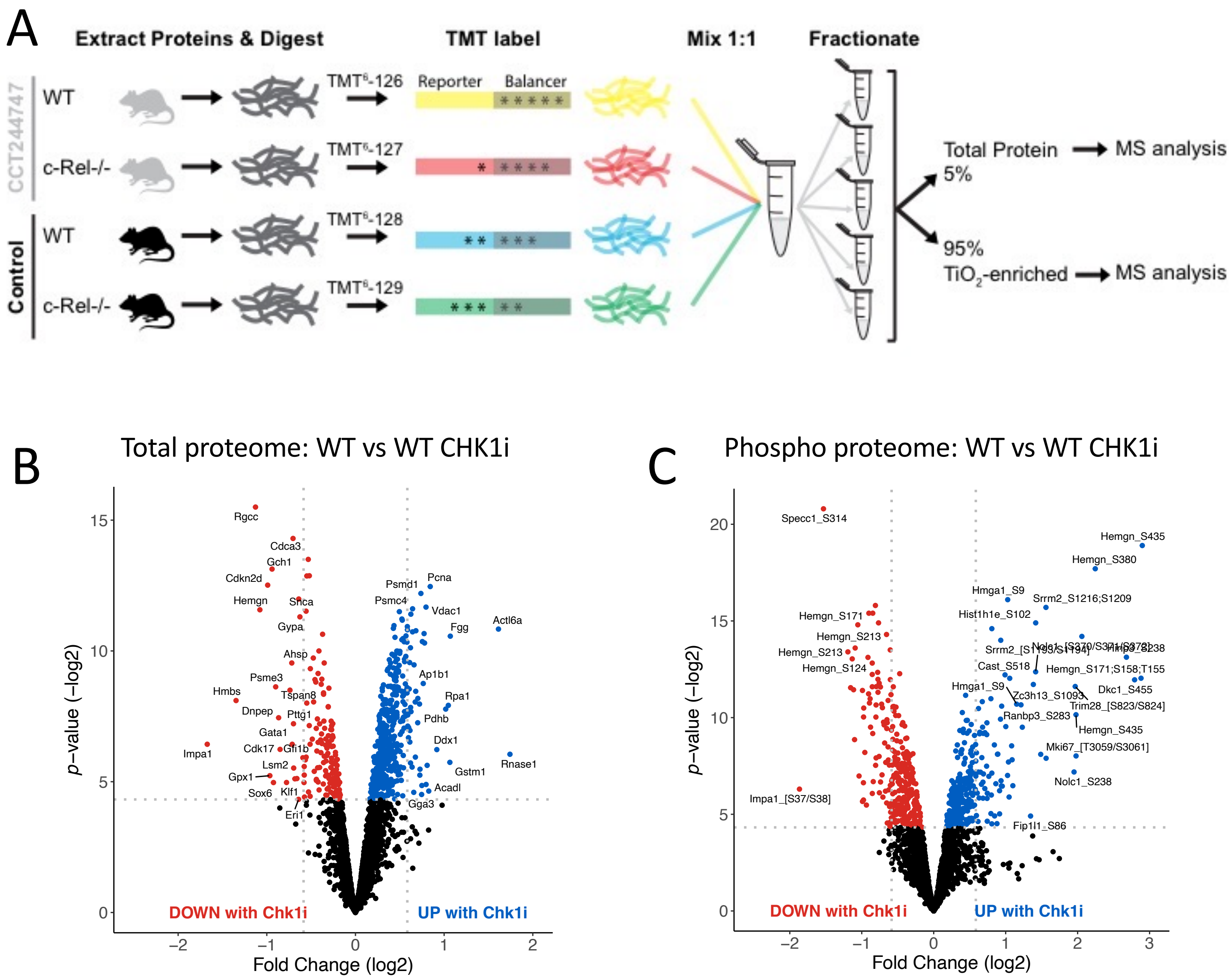

Hunter et al., Figure S3

A

Ratio	#Phosphopeptides significant		#Proteins significant	
	p value ≤ 0.05	*q* value ≤ 0.1	*p* value ≤ 0.05	*q* value ≤ 0.1
WT_Control/WT_Chk1i	625	165	622	0
cREL_Control/cREL_Chk1i	89	0	162	0
cREL_Control/WT_Control	1106	830	966	634
cREL_Chk1i/WT_Chk1i	691	31	943	394

B

Phosphosite overlap

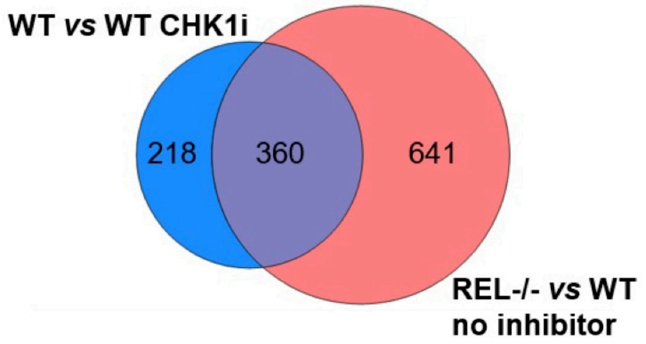

C

Total proteome overlap

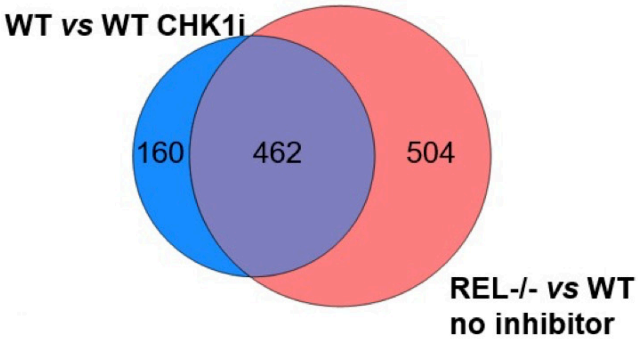

D

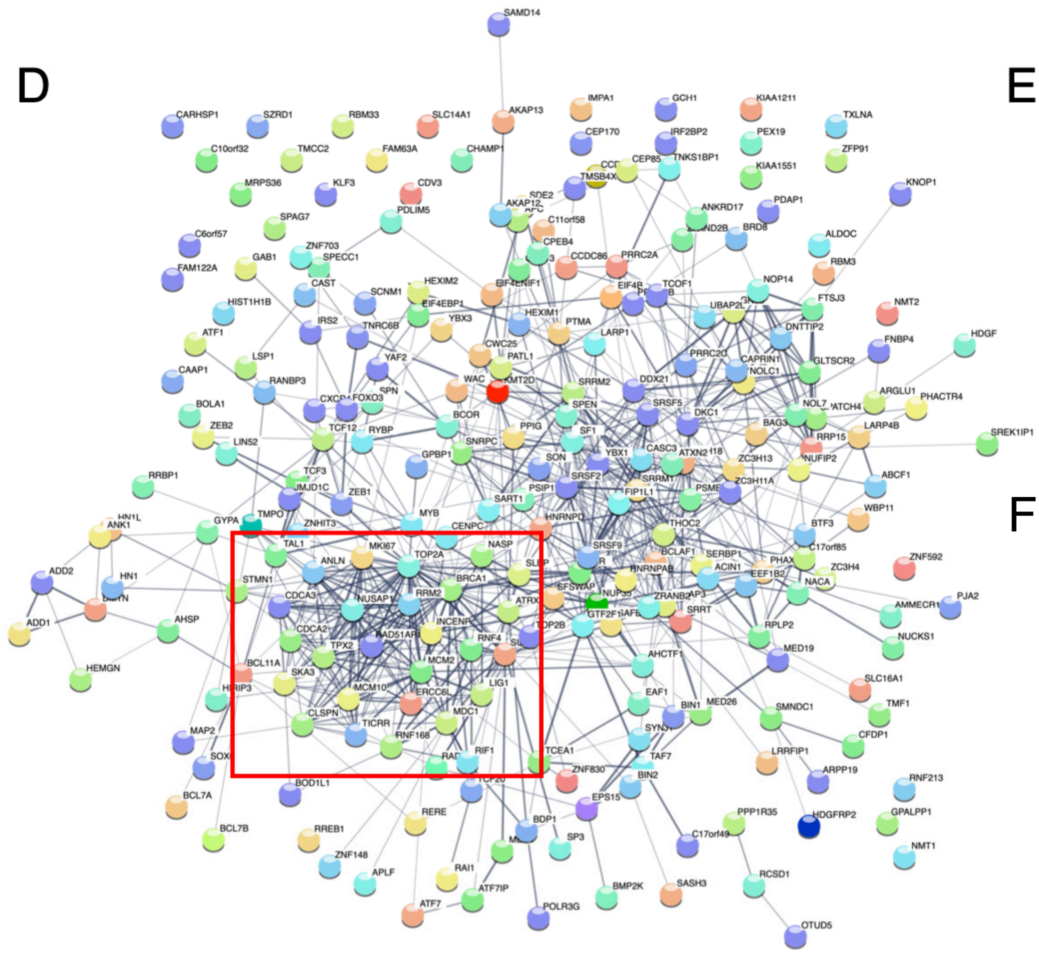

E

CHK1 added
(high confidence network)

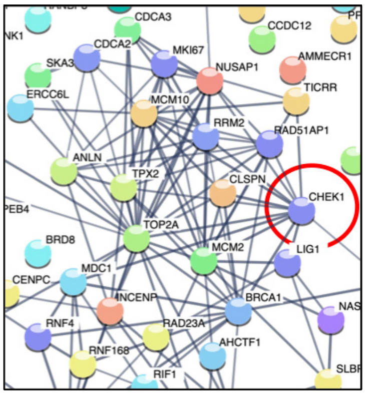

F

No CHK1 added
(high confidence network)

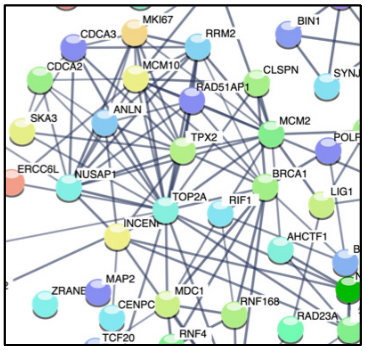

Hunter et al., Figure S4

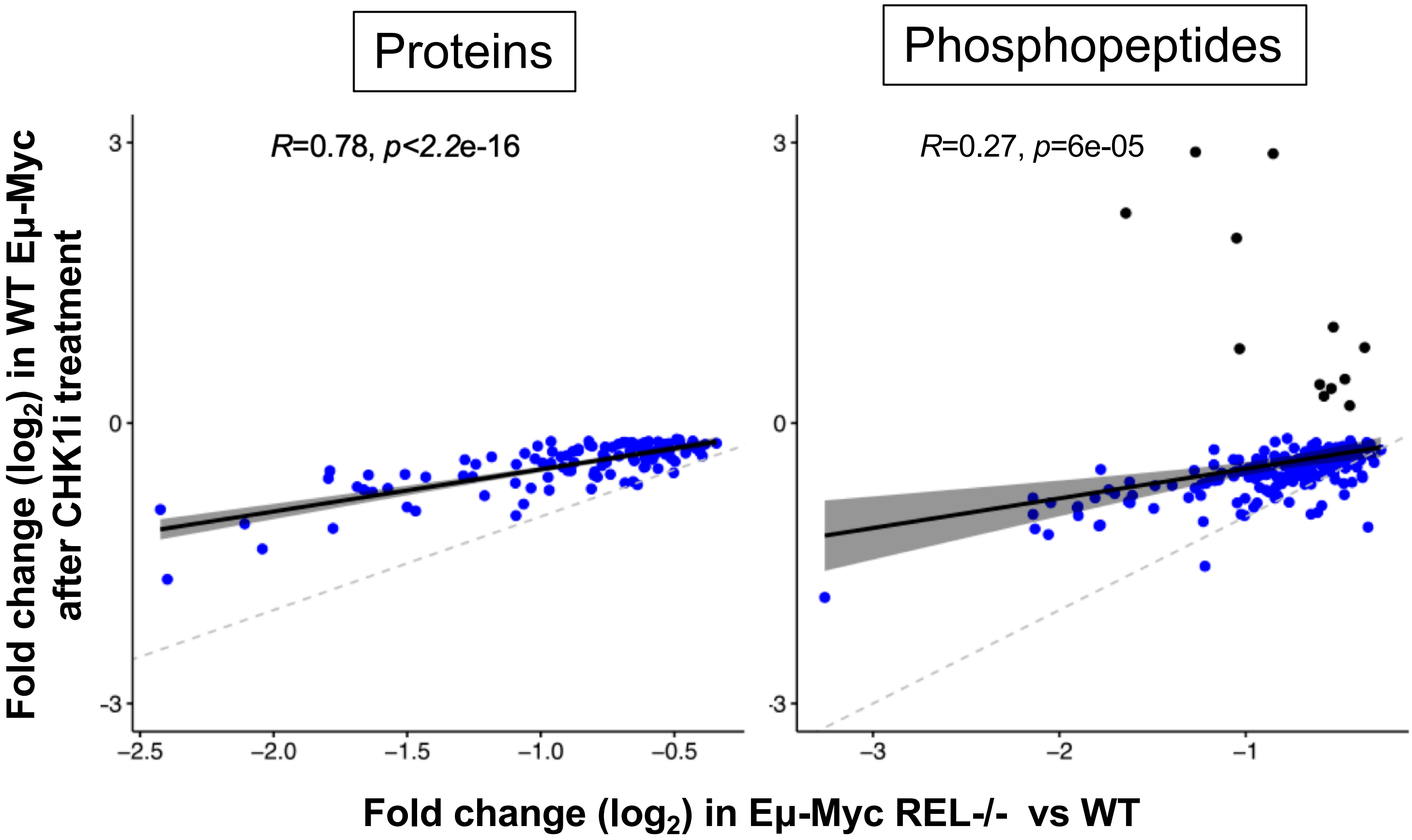

Hunter et al., Figure S5

A

Symbol	log2FC	pAdj
ATR	-0.67	1.29E-08
ATRIP	-0.59	2.41E-04
CDC25A	-1.22	6.15E-11
CDC25C	-2.64	9.20E-19
CDC45	-2.00	3.01E-14
CDC6	-2.08	1.22E-09
CDC7	-1.70	3.51E-19
CDK2	-1.13	1.78E-24
CHEK1	-2.25	2.66E-12
CLSPN	-1.95	4.44E-09
DBF4	-1.49	5.30E-15
HUS1	-0.86	3.80E-04
MCM10	-2.04	3.90E-08
MCM2	-1.44	1.63E-07
MCM3	-1.68	2.20E-11
MCM4	-1.54	8.92E-13
MCM5	-1.64	2.62E-10
MCM6	-1.79	5.77E-13
MCM7	-1.60	9.26E-12
MCM8	-1.50	3.46E-11
ORC1	-1.28	6.30E-02
ORC2	-1.59	4.84E-29
ORC3	-0.56	4.23E-12
ORC4	-0.85	4.13E-06
ORC5	-0.99	4.22E-29
ORC6	-1.51	1.95E-13
RAD1	-0.79	7.23E-10
RAD17	-0.33	2.74E-05
RAD9A	-0.02	8.77E-01
RAD9B	0.51	1.95E-02
RFC2	-1.03	1.08E-13
RFC3	-1.21	1.16E-09
RFC4	-1.87	9.96E-14
RFC5	-1.35	1.48E-09
RPA1	-0.77	7.21E-09
RPA2	-1.28	6.32E-09
RPA3	-1.38	1.60E-09

B

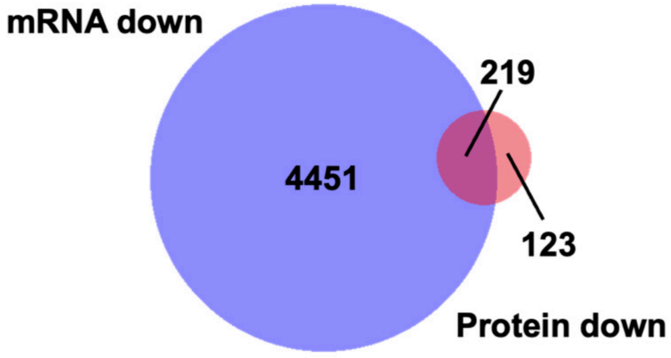

Overlap between downregulated protein encoding transcripts (RNA Seq) vs down regulated proteins (proteomics) in REL-/- vs WT Eμ-Myc lymphomas

C

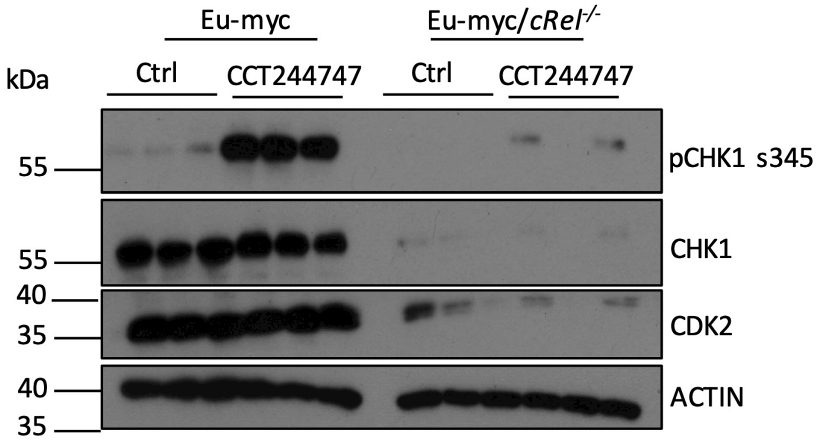

D

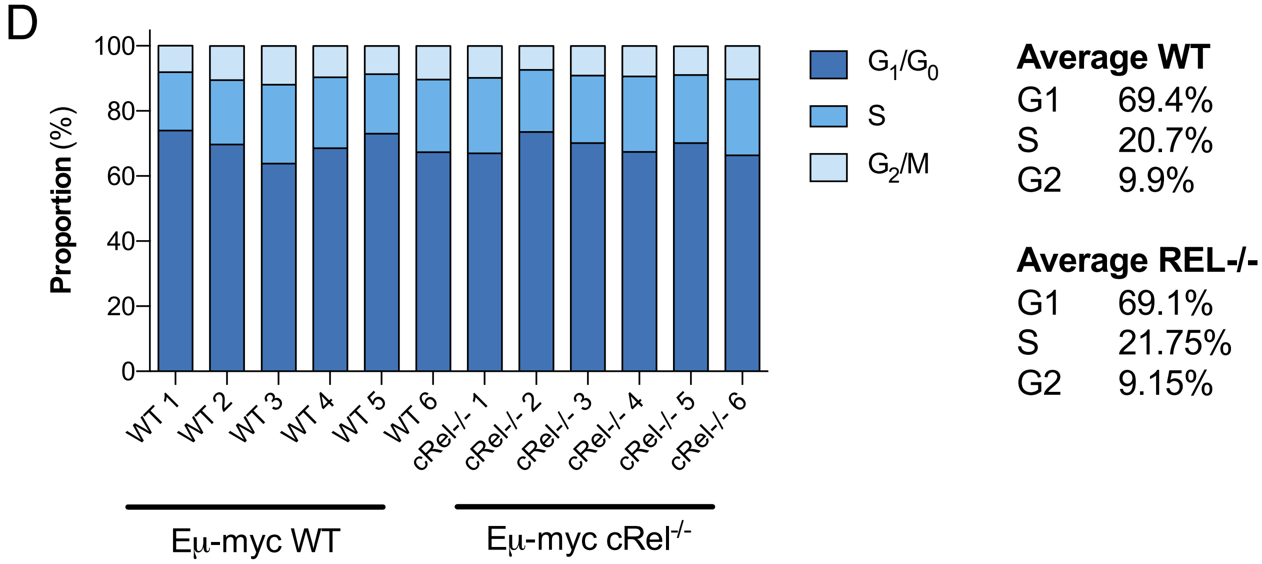

Hunter et al., Figure S6

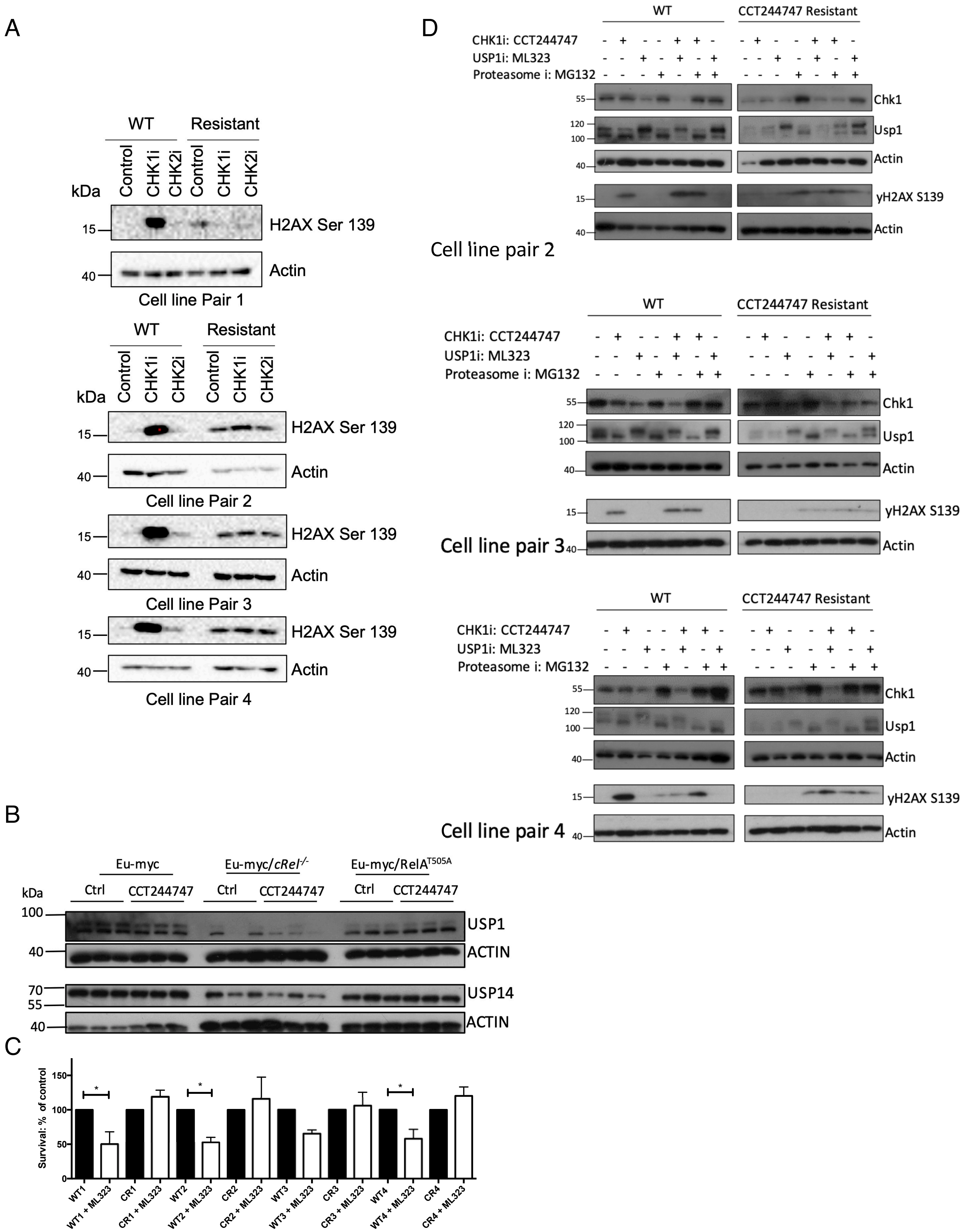

Supplement: Supplementary Material [file BCJ-479-2063-s1.pdf]
